# Direct single cell-type gene expression analysis in peripheral blood: novel ratio-based gene expression biomarkers using 2 novel monocyte reference genes (*PSAP* and *CTSS*) for detection of bacterial infection

**DOI:** 10.1093/hmg/ddaf103

**Published:** 2025-06-23

**Authors:** Nelson L S Tang, Tsz-Ki Kwan, Dan Huang, Suk-Ling Ma, Kwong-Sak Leung

**Affiliations:** Department of Chemical Pathology, and Li Ka Shing Institute of Health Science, Faculty of Medicine, The Chinese University of Hong Kong, Hong Kong SAR, China; Cytomics Limited, Hong Kong Science Park, Hong Kong SAR, China; Hong Kong Branch of CAS Center for Excellence in Animal Evolution and Genetics and KIZ/CUHK Joint Laboratory of Bioresources and Molecular Research in Common Diseases, Hong Kong SAR, China; Functional Genomics and Biostatistical Computing Laboratory, CUHK Shenzhen Research Institute, Shenzhen, China; Cytomics Limited, Hong Kong Science Park, Hong Kong SAR, China; Southern University of Science and Technology, Shenzhen 518055, China; School of Arts and Humanities, Tung Wah College, Hong Kong SAR, China; Department of Computer Science and Engineering, Faculty of Engineering, The Chinese University of Hong Kong, Hong Kong SAR, China

**Keywords:** Single cell-type gene expression, Monocyte, Bacterial infection, blood, qPCR, DIRECT LS-TA, Ratio-based biomarker

## Abstract

**Background:**

To determine single-cell-type gene expression in peripheral blood (PB) requires either prior cell sorting or single-cell RNA sequencing. We developed a novel ratio-based biomarker (RBB) called Direct Leukocyte Subpopulation-Transcript Abundance (DIRECT LS-TA) that allows quantification of monocyte-specific gene expression directly from PB without cell sorting.

**Methods:**

DIRECT LS-TA leverages proportional cell counts and differential gene expression profiles among leukocyte subpopulations to identify monocyte-informative genes. Using a new ICEBERG plot (Figure 1) based on a mathematical model of cell-mixture gene expression, we shortlisted genes with 2.5-fold higher expression in isolated monocytes compared to PB, indicating > 50% of transcript contribution by monocytes alone. *PSAP* and *CTSS* were identified as monocyte informative reference genes with low biological variation. Using one of them as the denominator, another monocyte informative target gene is used as the numerator to derive the RBB. The method was validated for detection of host response towards bacterial infection across multiple datasets.

**Findings:**

Over 50 monocyte-informative genes were identified, including immune response genes such as *VNN1, IL1B, NLRC4* and *IFI44L*. DIRECT LS-TA results showed excellent correlation with gold standard isolated monocyte expression (R^2^ = 0.55–0.97). *VNN1* RBB showed consistent upregulation across five datasets (median 2.7-fold, *P* < 10^−8^) with good diagnostic performance (AUC = 0.84–0.99). Other genes including *NLRC4*, *CYP1B1* and *NFKBIZ* were also useful biomarkers.

**Conclusion:**

DIRECT LS-TA provides a reliable way of quantification of monocyte-specific gene expression from PB without the need of cell sorting and demonstrated potential use for rapid infection detection and antibiotic stewardship.

## Introduction

Expression levels or transcript abundance (TA) of genes in peripheral blood cells serve as important biomarkers. However, many clinical applications of quantification of TA in peripheral blood (PB) samples were performed on cell mixture samples such that the TA results represented the summation of TA of all the various cell types of leukocytes. And no information of TA of a specific leukocyte cell-type or leukocyte subpopulation (LS such as B lymphocytes or monocytes) can be obtained directly from the bulk RNA quantification in PB.

On the other hand, TA of genes of a specific LS are the preferred biomarkers. In order to obtain TA of a specific LS, the current state of the art requires prior cell sorting to isolate the specific LS from PB before quantification of TA by quantitative PCR (qPCR) or digital PCR (dPCR). Latest technology of single-cell RNA sequencing can also obtain TA of a specific LS even at the level of every single cell [1, 2]. However, both methods are either not applicable or affordable in common clinical use. Cell sorting/isolation is too laborious and tedious to run in a routine hospital laboratory. Single-cell RNA sequencing is too expensive to use for every patient admitted for investigation of febrile illness, for example. These procedures are not practical in the setting of a clinical service laboratory. A straight-forward laboratory protocol to obtain TA for genes of interest of a specific LS in PB is needed. We described a prototype of a direct method to obtain single cell-type specific TA of B lymphocytes [3, 4]. Here, the same logic is applied to target monocyte with the objectives of determining TA in monocytes directly from PB without prior monocyte separation.

Peripheral blood mononuclear cells (PBMC) is a commonly used PB sample type which is composed of lymphocytes T cells, B cells, monocytes and natural killer cells. The various LS are present in given ranges of cell proportions. For example, B lymphocytes typically comprise about 5% of leukocytes in PB. Monocytes account for 10% to 30% of leukocytes in PBMC. The direct single cell-type specific transcript abundance approach (DIRECT LS-TA) utilises the given cell proportion of the specified cell-type to shortlist a list of genes whose transcripts in PB are produced predominantly by that specified single cell-type. We have succeeded in applying DIRECT LS-TA method to analyse B cells TA response as an early biomarker of seroconversion after vaccination [4]. With the proof of principle method development for the B lymphocytes having a cell-count proportion as low as 5%, this DIRECT LS-TA approach would be even more powerful when applied to cell-types present in a higher cell-count proportion. Monocytes are present at a higher cell proportion in PBMC and a given figure of 20% was used for method development in this paper. A more detailed description of how to shortlist monocyte informative genes is provided in the materials and methods section.

Other studies attempted to address the cell mixture problem from a different perspective. For example, deconvolution methods [5–7] had been developed to determine the cell-count proportion of each LS cell-type in PB cell-mixture samples but most of these methods cannot obtain gene TA in an individual sample as TA of a gene is commonly assumed as fixed group-wise values for patients and controls. The failure to capture gene TA variations for an individual and requirement of TA data of the whole genome are common limitations of deconvolution methods. Recently, there are also new bioinformatic approaches to obtain single-cell-type specific gene expression for individual samples in bulk RNA sequencing data of cell-mixture samples by using AI assisted and Bayesian statistical methods [8–13]. Again, these methods require input of TA data of the whole genome which are labour intensive and expensive. With the motivation of developing a method that can be readily translated for clinical application which requires only a quantification of handful of genes, we carried out this bioinformatic investigation to determine the feasibility of direct determination of monocyte TA in PB. In this study, we aimed to develop the monocyte DIRECT LS-TA method to obtain TA of monocytes in patients with bacterial infections.

## Results

### Shortlisting of potential monocyte cell-type specific (informative) genes

**Figure 1:**
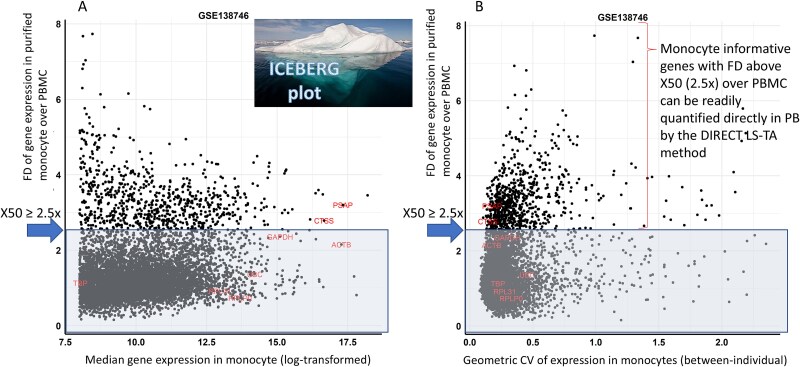
Two versions of ICEBERG plots. (A). An ICERBERG plot showing genes expressed in monocytes and PBMC to shortlist monocyte informative genes. The scatter plot shows the expression fold difference (FD) of genes between isolated monocytes and the corresponding PBMC sample (values on the y-axis, units as folds). Genes with FD exceeding the X50 threshold of 2.5x are candidate monocyte informative genes as they are predominantly produced by monocytes in PB. The x-axis shows the expression level in monocytes as log normalised counts. Typical housekeeping genes (e.g. *RPL31, ACTB, RPLP0, GAPDH, TBP* and *UBC*) are labelled but their FD are all below 2.5x, therefore, they are not useful in the DIRECT LS-TA assay for direct quantification of monocyte gene expression in PB. The 2 monocyte informative reference genes (*PSAP* and *CTSS*) are also shown in the figure. Data source is GSE138746. ICEBERG is used as an analogy here with the above-water part representing those monocyte informative genes above the shade. (B). Another format of the ICEBERG plot showing the FD against between-individual variation of gene expression in terms of geometric CV. Monocyte informative reference genes should have a low level of biological variation (those positioned to the left end on x-axis in the chart). The 2 monocyte informative reference genes (*PSAP* and *CTSS*) are labelled in the figure together with conventional housekeeping genes known to have low biological variations.

Based on the workflow (See Method Section) and mathematical framework (Supplementary materials), it is possible to identify monocyte informative genes by using the fold difference (FD) of gene expression between purified monocyte and corresponding PB samples. A FD higher than 2.5 is required for monocyte informative genes. Monocyte informative genes need to have at least 2.5x higher expression in purified monocyte compared to genes in PBMC. This FD is plotted against the median expression level (log-transformed) in Fig. 1A and against the geometric coefficient of variation (CV) in Fig. 1B. These plots are called ICEBERG plots as genes expressed by monocytes with a higher FD over PBMC would have their monocyte specific gene expression be more discernible in PB than other genes with lower FD. Therefore, these monocyte informative genes appear as the above-water part of the ICEBERG, while other genes are represented as the below-water part of the ICEBERG and cannot be readily quantified.

In Fig. 1B, X-axis of ICEBERG plots shows the CV of between-subject variation of gene expression. It is used to select the monocyte informative reference gene which is used as the denominator in the DIRECT LS-TA RBB. The two genes showing the least CV, *PSAP* and *CTSS* are shown in Fig. 1B. Their CV is comparable to other conventional housekeeping gene (HKG) which are also labeled in Fig. 1, however, all HKGs have a FD below 2.5x and they cannot be used in the DIRECT LS-TA method.

### Correlation between monocyte DIRECT LS-TA RBBs in PB cell-mixture samples and gene expression of isolated monocytes

50 monocyte cell-type specific (informative) genes are listed in Table 1. These monocyte cell-type specific genes span various cell signaling pathways including genes like *VNN1, NFKBIZ, IFI44L and IL1B*.

**Table 1:** List of monocyte cell-type specific informative target genes and 2 reference genes (*CTSS* and *PSAP*).

Reference and Target gene	The 90^th^ percentile value of the fold of expression in monocytes vs. peripheral blood or PBMCs	Geometric Coefficient of variation	Correlation between the Monocyte DIRECT LS-TA biomarker obtained directly from peripheral blood samples and the gold standard (expression level of target genes in isolated monocytes) (coefficient of determination, r^2^)^b^	Database source showing the best correlation
*CTSS*	2.7x	0.14	NA^a^	GSE138746
*PSAP*	3.1x	0.18	NA^a^	GSE138746
*ASGR2*	3.6x	0.69	0.79	GSE138746
*ATF3*	3.8x	4.44	0.64	GSE138746
*CALHM6*	2.7x	1.14	0.78	GSE138746
*CD163*	3.4x	0.63	0.85	GSE138746
*CD36*	3.4x	0.31	0.64	GSE138746
*CDKN1A*	2.8x	1.59	0.81	GSE138746
*CES1*	3.5x	1.02	0.85	GSE138746
*CLEC12A*	3.1x	0.50	0.73	GSE138746
*CRISPLD2*	3.6x	0.48	0.72	GSE138746
*CXCL10*	10.1x	2.28	0.84	GSE60424
*CYP1B1*	3.9x	0.59	0.80	GSE138746
*CYP27A1*	4.2x	0.48	0.65	GSE138746
*EREG*	6.4x	2.44	0.66	GSE138746
*FOS*	4.0x	2.09	0.82	GSE138746
*GADD45B*	2.5x	0.96	0.70	GSE138746
*IER2*	3.2x	1.92	0.74	GSE138746
*IFI30*	3.4x	0.50	0.60	GSE60424
*IFI44L*	3.2x	3.01	0.95	GSE60424
*IFITM3*	2.6x	1.70	0.87	GSE138746
*IL1B*	3.7x	2.71	0.80	GSE138746
*KLF10*	2.5x	0.83	0.83	GSE138746
*LILRA5*	3.3x	0.33	0.55	GSE114407
*LYZ*	3.4x	0.46	0.69	GSE138746
*MAFB*	4.1x	0.77	0.73	GSE138746
*MARCO*	3.4x	0.69	0.75	GSE138746
*MERTK*	3.3x	0.70	0.60	GSE138746
*MYOF*	2.8x	0.64	0.68	GSE138746
*NAIP*	3.0x	0.61	0.66	GSE138746
*NFKBIA*	3.8x	1.27	0.55	GSE114407
*NFKBIZ*	2.7x	1.02	0.60	GSE114407
*NLRC4*	2.9x	0.54	0.74	GSE138746
*NR4A1*	2.5x	3.72	0.82	GSE138746
*NRG1*	4.2x	1.32	0.85	GSE138746
*PFKFB3*	3.2x	0.97	0.68	GSE114407
*RHOB*	3.7x	1.24	0.72	GSE138746
*RNF144B*	2.7x	0.63	0.75	GSE138746
*RPH3A*	3.9x	1.41	0.73	GSE138746
*SCO2*	3.0x	0.80	0.66	GSE138746
*SGK1*	3.3x	1.03	0.82	GSE138746
*SHTN1*	3.5x	0.47	0.65	GSE138746
*SIGLEC1*	3.8x	3.09	0.94	GSE138746
*SULT1A1*	2.5x	0.59	0.80	GSE138746
*TCN2*	3.9x	0.65	0.65	GSE138746
*TLR7*	2.5x	0.68	0.66	GSE138746
*TMEM176A*	3.9x	4.91	0.92	GSE138746
*TMEM176B*	3.8x	5.11	0.97	GSE138746
*VNN1*	4.0x	1.14	0.80	GSE138746
*WARS1*	2.5x	0.68	0.72	GSE138746

^b^R^2^ values of these listed monocyte informative target genes are statistically significant (*P* < 1x10^−5^, equivalent to *P* < 0.001 after Bonferroni correction).

^a^ *CTSS* and *PSAP* are used as denominator genes in the RBB, so they do not have correlation results to compare with gold standard method.

The performance of using *PSAP* or *CTSS* as the reference gene (denominator of the DIRECT LS-TA RBB) was evaluated in datasets containing gene expression data of both isolated monocytes and cell-mixture samples (PBMC). Gene expression levels in the isolated monocyte samples are the gold standard in this analysis. The ability of DIRECT LS-TA RBB in PBMC to reflect the gold standard expression levels in isolated monocytes was evaluated by Pearson’s correlation of the 2 expression results. A correlation coefficient (r) > 0.7 was defined as the required level of correlation between the 2 gene expression results (i.e. R^2^ > 0.5).

Supplementary Fig. S1 shows the results of a typical workflow of this correlation evaluation between the 2 gene expression results (isolated monocyte and DIRECT LS-TA in PB). For example, *LYZ* is selected as the target gene and its expression level in monocytes is of interest. The gold standard *LYZ* expression level is that in the isolated monocytes and was normalized to a conventional housekeeping gene (*B2M*) [14]. The results were plotted on the x-axis as log (*LYZ*  _(monocytes)_/*B2M*_(monocytes)_), equivalent to log *LYZ*  _(monocytes)_—log *B2M*  _(monocytes)_ which represents the gold standard monocyte specific expression level of *LYZ*. The new DIRECT LS-TA assay of *LYZ* is a RBB using gene expression data quantified in WB, i.e. log (*LYZ*_(WB)_/*CTSS*_(WB)_) or equivalent to log *LYZ*_(WB)_—log *CTSS*_(WB)_. In this example, *CTSS* was used as the monocyte informative reference gene and as the denominator gene in the new RBB in the PB (cell-mixture) sample.

As shown in Table 1, shortlisted monocyte cell-type informative genes had good correlation with the gold standard monocyte gene expression evident by R^2^ (coefficient of determination) of 0.5 or above in more than one dataset in Table 3. The observed R^2^ ranged from 0.55 to 0.97 which indicated the ability of DIRECT LS-TA, a RBB in PB to reflect monocyte specific gene expression of these target genes. The results supported that for these shortlisted monocyte informative target genes, their monocyte specific gene expression is readily discernible in PB by this method. Next, these RBB were evaluated for detecting the host’s response to bacterial infection.

**Figure 2:**
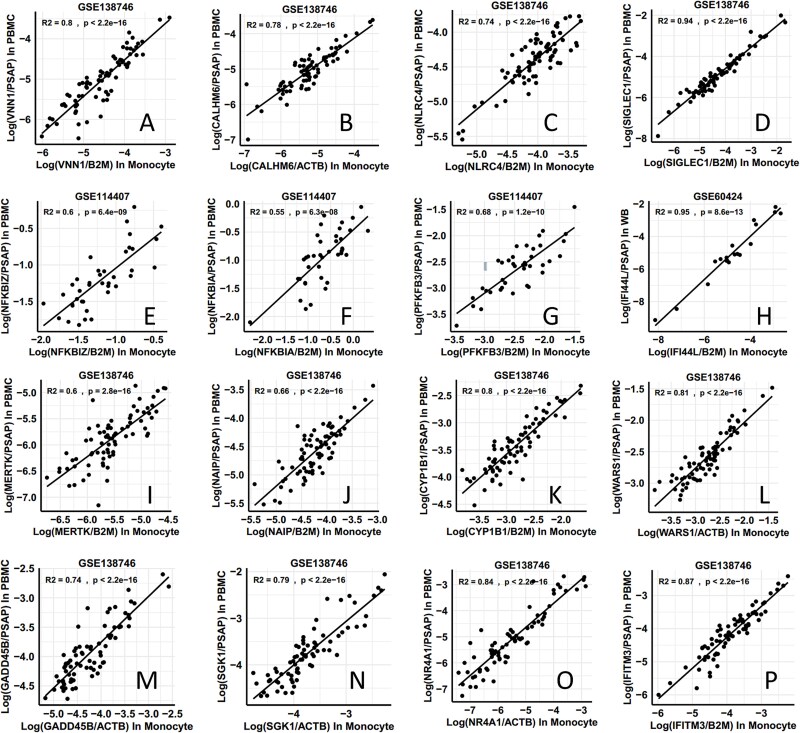
The correlation between monocyte DIRECT LS-TA for monocyte informative target genes measured in PB samples and expression levels of the same target genes in isolated monocytes obtained by the traditional method, using *PSAP* as a monocyte informative reference gene. (A) Shows the *VNN1* gene expression in monocytes determined by the method of DIRECT LS-TA and the traditional cell isolation method. The Y-axis is the ratio of log (*VNN1*_(PBMC)_/*PSAP*_(PBMC)_) determined directly from PB samples (i.e. monocyte DIRECT LS-TA biomarker of *VNN1* gene). The X-axis is the gold standard, using the traditional method to detect *VNN1* expression after purification of monocytes, and a conventional housekeeping gene (*B2M*) is used for normalization, i.e. log (*VNN1*_(monocytes)_/*B2M*_(monocytes)_). As shown in (A), there is a good correlation between the two. Evaluation of the correlation for other monocyte informative genes using DIRECT LS-TA in peripheral blood is shown in (B-P), where the genes are *CALHM6, NLRC4, SIGLEC1, NFKBIZ, NFKBIA, PFKFB3, IFI44L, MERTK, NAIP, CYP1B1, WARS1, GADD45B, SGK1, NR4A1* and *IFITM3* respectively. Dataset accession numbers for data sources are shown above (A-P). All monocyte informative genes show a high and statistically significant correlation (R^2^ > 0.5).

Figures 2 and Supplementary Fig. S2 show the extent of correlation between gold standard gene expression results in isolated monocyte (X axis) and the DIRECT LS-TA method using gene expression measured in PB (Y axis) with *PSAP* and *CTSS* as the denominator genes, respectively. For most genes, results in the GSE138746 dataset were shown. Some target genes were not present in that dataset or had much higher correlation in another dataset and they were shown instead.

Both *PSAP* and *CTSS* were investigated if their expression in monocyte were changed by clinical conditions among 78 patients with rheumatoid arthritis in the dataset. As shown in the supplementary material, expression of both genes was not affected by factors like sex, age, body height, use of alcohol, total leukocyte count, treatment response and presence of various auto-antibodies.

### Standardization of DIRECT LS-TA RBB results and use as biomarkers for bacterial infection

Using the WB gene expression results in the GSE154918 dataset [18], monocyte DIRECT LS-TA of *VNN1* gene is calculated as (Monocyte DIRECT LS-TA of *VNN1*) = (*VNN1*_(WB)_)/(*PSAP*_(WB)_) or its log transformation, log(*VNN1*_(WB)_)—log(*PSAP*_(WB)_). This is similar to delta CT (ΔCT) in qPCR experiments.

To convert to fold change against a healthy reference individual, or delta–delta CT (ΔΔCT) in qPCR experiments, multiples of median (MoM) of monocyte DIRECT LS-TA is obtained by subtracting monocyte DIRECT LS-TA results of patients from that of the median of the control group (refer to the materials and methods). By setting the median of log Monocyte DIRECT LS-TA of the control (healthy) group to zero, a MoM of log DIRECT LS-TA of each sample is similar to the delta–delta CT values in qPCR or dPCR experiments. It represents the activation (fold change) of target gene over healthy controls on log scale. This is used as a biomarker for disease risk and evaluated for diagnostic performance.

**Figure 3:**
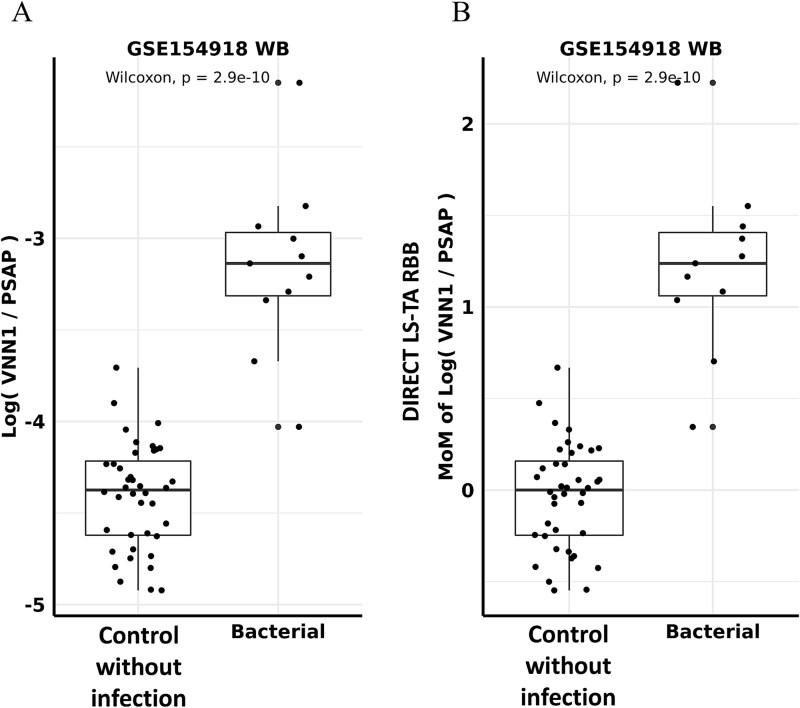
MoM conversion of direct monocyte LS-TA of the *VNN1* gene in the GSE154918 dataset. (A) Shows the log (monocyte DIRECT LS-TA of the *VNN1* gene) (i.e. log (*VNN1*_(WB)_/*PSAP*_(WB)_) of the control group and the uncomplicated bacterial infection group, respectively. (B) Shows the results after converting the log (*VNN1*_(WB)_/*PSAP*_(WB)_) in (A) to a multiple of median (MoM). MoM results is higher in the bacterial infection group. It corresponds to a ΔΔCT result of 1.7 cycles when DIRECT LS-TA would be adapted into a qPCR platform.

Figure 3 shows the conversion of Monocyte DIRECT LS-TA of *VNN1* from delta CT equivalent (Fig. 3A) to delta–delta CT (ΔΔCT) equivalent MoM values (Fig. 3B). The sample distribution shown in Fig. 3B had no actual change when compared to the sample distribution in Fig. 3A. The advantage of using the multiple of median (MoM, Fig. 3B) was that the median of the normal control group was set to zero, which allowed comparison of fold changes in DIRECT LS-TA (monocyte specific gene expression) in disease across databases. MoM results can be converted to the expected ΔΔCT results when DIRECT LS-TA is adapted to qPCR or dPCR platforms. MoM was 1.2 in Fig. 3B which represented a fold change of e^1.2 = 3.3 fold. The corresponding ΔΔCT result in qPCR is 1.7 cycles.

**Figure 4:**
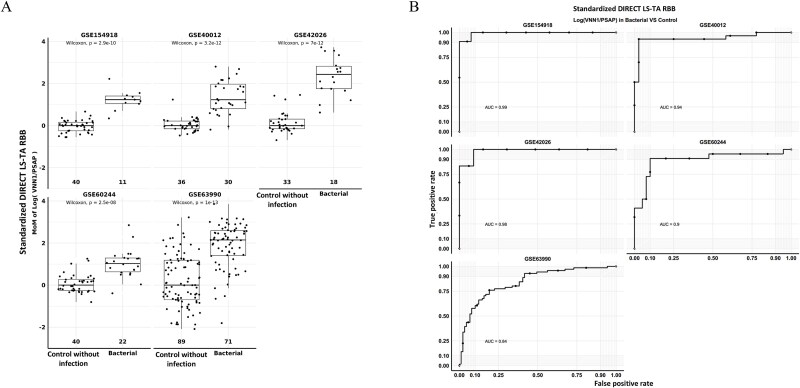
(A) Analysis of the MoM of the monocyte DIRECT LS-TA *VNN1* in the control group and the bacterial infection group. (B) Receiver operating characteristic (ROC) curve analysis of the discriminative performance of the monocyte DIRECT LS-TA *VNN1* in the bacterial infection group. (A) Shows direct LS-TA RBB of VNN1 gene was analysed in five other datasets (GSE154918, GSE40012, GSE42026, GSE60244, and GSE63990). The numbers above the X-axis represent the number of people in the control group and the bacterial infection group, respectively. In each dataset, the MoM results on the Y-axis are log-transformed result of DIRECT LS-TA results. (B) Shows the diagnostic performance of the monocyte DIRECT LS-TA assay of the target gene *VNN1* was performed by ROC analysis. In all 5 datasets, the results of area under curve ranged from 0.84 to 0.99.

Figure 4A shows the MoM of log (*VNN1*_(WB)_/*PSAP*_(WB)_) or Monocyte DIRECT LS-TA *VNN1* in healthy controls and bacterial infection patients in the discovery dataset GSE154918 and four other replication datasets. Conceptually, the MoM is related to delta–delta CT (ΔΔCT) in qPCR and indicates that there were more than 2 folds increase in Monocyte DIRECT LS-TA *VNN1* in most datasets. The GSE60244 dataset had the least activation but showed a median increase by more than 2 folds. The differences in Monocyte DIRECT LS-TA *VNN1* were highly significant in all datasets by Wilcoxon non-parametric tests (*P* values from 2.5x10^−8^ to 1x10^−13^). ROC analysis was also performed on the discovery dataset (AUC = 0.99) and in the replication datasets, which returned AUCs ranging from 0.84 to 0.98 (Fig. 4B).

Other monocyte informative genes were also activated in patients with bacterial infection including *NLRC4, CYP1B1, PFKFB3, LILRA5, NFKBIA*, and *NFKBIZ*.

**Figure 5:**
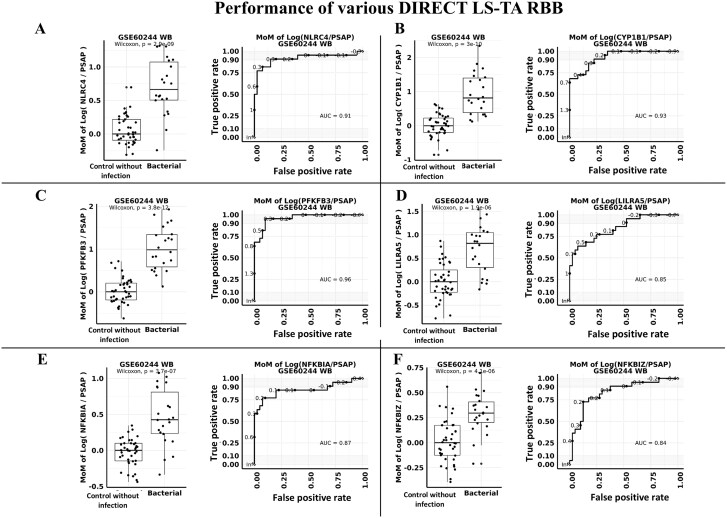
Analysis of monocyte DIRECT LS-TA assays for six additional target gene (i.e. *NLRC4, CYP1B1, PFKFB3, LILRA5, NFKBIA*, and *NFKBIZ* respectively) using *PSAP* as reference gene and their receiver operating characteristic (ROC) curve analysis of the discriminative performance in differentiating uncomplicated bacterial infection. (A-F) Show the diagnostic performance of six additional target genes of monocytes, the expression of which are activated after bacterial infection. Here, the monocyte DIRECT LS-TA is calculated using *PSAP* as a reference gene. For each gene, the difference between MoM of monocyte DIRECT LS-TA in peripheral blood between the control group and the bacterial infection group is shown by boxplots (left). The right panel shows results of ROC analysis.

Figure 5 showed the MoM of Monocyte DIRECT LS-TA of these additional genes and their diagnostic performance in ROC analysis. Wilcoxon group-wise *P* values ranged from 1.9x10^−6^ (DIRECT LS-TA *LILRA5*) to 3.8x10^−12^ (DIRECT LS-TA *PFKFB3*). AUC of MoM of DIRECT LS-TA of these 6 additional target genes was over 0.8.

Other than *PSAP*, *CTSS* could be used as the denominator gene of the RBB, Monocyte DIRECT LS-TA. Supplementary Fig. S3 shows the evaluation of Monocyte DIRECT LS-TA using *CTSS* as the denominator gene. Similarly, Wilcoxon group-wise *P* values ranged from 1.3x10^−6^ (DIRECT LS-TA *NFKBIZ*) to 6.9x10^−12^ (DIRECT LS-TA *PFKFB3*). AUC of MoM of DIRECT LS-TA of these 6 additional target genes was over 0.8. The results suggested that both denominator genes (*PSAP* and *CTSS*) produced similar results and performance in terms of detection of host response towards bacterial infection.

## Discussion

Biomarkers of host response to infection have great clinical applications in triage of patients with fever coming to the clinic or emergency department. Early differentiation of patients with potential bacterial infection is important so that they can be managed promptly and necessary samples are collected for bacteriological investigation in time. Traditional bacterial culture takes days to complete. Even the latest state-of-the-art method using an ultra-rapid pathogen ID assay takes at least 12 hours [19].

Presently, only C-reactive protein (CRP) and procalcitonin (PCT) are in routine clinical use. Both are serum protein markers so they do not convey any cell-type specific host response information, but just represent an overall systemic host response to infection. Therefore, there are overlapping responses to different types of infection. For example, both viral and bacterial infections lead to an elevation of CRP; thus in some patients making such differentiation is difficult by using these protein biomarkers.

PB is a cell-mixture sample of leukocytes of various subpopulations or cell-types (e.g. monocyte, granulocytes and lymphocytes). Proportional cell counts of these subpopulations are useful in the differential diagnosis of fever. For example, the granulocyte percentage increases in bacterial infections and lymphocyte count increases in viral infections. However, the between-individual variations of these cell count proportions are large and it is difficult to get cutoff values to make the differential diagnosis (Table 2).

**Table 2:** Pros and cons of different approach.

**Methods/Approach**	**Information obtained**	**Limitations**
Serum CRP or Procalcitonin	Serum protein biomarker	Systemic response of host No cell-based information
Complete Blood Counts	Cell count proportions of leukocyte subpopulations (e.g. monocytes, lymphocytes)	Cell counts only No Cellular information
DEG of PB samples (e.g. RNA-seq of PAXgene Blood RNA Tube)	Summative gene expression of all cell types (also known as bulk gene expression data)	Results confounded by both change in cell counts and gene expression in leukocyte subpopulations
Deconvolution analysis of bulk gene expression data	Retrieve cell count proportions from bulk gene expression data	No information of single cell-type (subpopulation) gene expression of an individual in the dataset (also see below)
Machine learning devolution analysis	Retrieve cell-count proportions also infer single cell-type gene expression of individual	Require whole genome expression profiling to perform the calculation
Single cell RNA-seq of PB	Observe both cell count and gene expression data of every single cell in PB	Expensive to run Take days for RNA-seq and bioinformatics work
[Gold Standard] Isolation of single cell-type and measure gene expression	Most accurate approach to get TA of a single cell-type population	Manual and labour-intensive cell sorting

With the advance in molecular techniques to quantify gene expression, many researchers analyzed the TA in PB using microarray or RNA-sequencing. The expression of each gene is statistically analyzed one by one, and then the genes with the greatest expression difference between different groups are identified as biomarkers. These gene expression biomarkers are also called differential expression genes (DEGs) [20–24]. This method ignores the confounding factor of the cell counts of various cell subpopulations and their variations in different diseases. Therefore, variations in these factors will weaken the effectiveness of DEG biomarkers in differentiating diseases. These studies showed that bacterial infection induced expression of a large battery of genes in PB. Two key factors cause DEG in PB: [1] A change in gene expression of one or more leukocyte cell-types and [2] A change in proportional cell counts. Both factors confound each other in the identification of DEG biomarkers in diseases. Therefore, a long list of DEGs were found and thus, many biomarker genes are included in subsequent assay [25–27]. Resulting in the limitation that these methods can only be carried out in a research laboratory setting requiring expensive equipment [28]. DEG confounded by cell count proportions in the cell-mixture sample of PB is not the optimal IVD to read out the host response.

Computation algorithms have been developed to deconvolute the cell-count proportion of each cell type presented in a PB sample using matrix deconvolution [5, 29]. However, these algorithms assumed the same expression profile for each cell-type for all subjects in a group. Only recently, methods have been developed to determine single cell-type gene expression for individual subjects in a dataset using machine learning methods [9]. However, all of these methods require the input of the gene expression data of the whole genome such as microarray data or RNA-sequencing data making these technologies unsuitable for everyday clinical use as they are still too expensive for routine use at the moment. Of course, the gold standard approach is to isolate monocyte from PB and measure gene expression of the target genes. However, it requires cell separation procedures which is tedious and technically challenging in a clinical laboratory setting. It is not practical to implement cell isolation procedures in routine hospital laboratory for the time being. The Pros and Cons of various methods are shown in Table 2.

In contrast, by using DIRECT LS-TA method, gene expression of a single cell-type (monocyte) in PB can be directly determined in every sample. In this study, it is used as a biomarker to detect bacterial infection using gene expression data available in the public datasets. This new DIRECT LS-TA RBB reflecting the TA of monocyte informative target genes that can be quantified directly in PB samples. The correlation of DIRECT LS-TA results and TA in isolated monocytes was very strong, R^2^ for some target genes were up to 0.9 or even more. DIRECT LS-TA results represent the average gene expression of a single cell-type, and therefore, is not confounded by any change of the cell count proportions in cell-mixture samples. Furthermore, this RBB method can be readily applied to clinical application as it only requires the use of qPCR or dPCR machines which are widely available nowadays in most clinical laboratories.

VNN1 encodes vanin-1, a membrane-associated enzyme with pantetheinase activity and it is involved in oxidative stress regulation, inflammation, and cellular metabolism. VNN1 is abruptly expressed in monocytes and neutrophils and involved in innate response [30]. Study showed that VNNI1 expression was upregulated at inflamed site induced by bacteria [31], suggesting its possible involvement in bacterial infection. NFKBIZ is another key marker identified in this study which might play a major role in responding to bacterial infection. NFKBIZ encodes IκBζ, is the nuclear member of the IκB family that modulates NF-κB transcriptional activity. It is known that NF-κB played an important role in inflammatory and immune response [32]. The expression of IκB in monocyte was highly increased after the stimulation of LPS in animal model [33]. All these evidence suggested that the key markers identified were associated with the anti-bacterial function in monocyte.

Antimicrobial resistance remains a global health challenge and over 4 million deaths were estimated to associate with bacterial microbial resistance in 2021 [34]. Accurate and timely discriminating diagnosis of bacterial infection is essential to reduce antibiotic misuse and overuse. In this article, we shortlisted activated target genes in monocytes during acute bacterial infection, including *VNN1*, *NLRC4, CYP1B1, PFKFB3, LILRA5, NFKBIA* or *NFKBIZ*. These monocyte informative target genes and *PSAP* or *CTSS* as the monocyte specific reference gene can be used as a new kind of RBB. Such monocyte DIRECT LS-TA assays are useful in differentiating bacterial infection. The high correlation of gene expression of these target genes in isolated monocytes and direct measurement of TA in PB without the need of cell sorting are unique features of Monocyte DIRECT LS-TA method. This technology is feasible to apply in clinical setting to provide a robust and accurate differential diagnosis of bacterial infection.

Our study was limited by confining to use publicly available gene expression datasets and having little control of the design of the original study e.g. case definition of bacterial infection and different platforms of gene expression quantification. Therefore, we confined our case selection to acute uncomplicated bacterial infection and excluded cases with systemic sepsis which is a heterogeneous condition [18]. Also, the sample size of the datasets was small (discovery dataset: 29 bacterial infection patients, Replication datasets: total 87 patients). Moreover, our study and results could only be used to discriminate bacterial infection as a group but not to identify the exact microbial pathogen involved. Other follow-up tests for example blood culture are needed to perform to identify the causative bacteria.

As the mathematical framework was developed from a PBMC dataset, this method is preferably applied to PBMC sample. It is now quite easy to obtain PBMC sample by using specialised blood collection tube. We did not come across recently RNA-sequencing dataset of PBMC in patients with bacterial infection, therefore, we extended to use data obtained from other PB samples (e.g. whole blood collected in PAX tube). As granulocytes will be confounding our results, we excluded genes that are expressed heavily by granulocytes from evaluation.

In conclusion, a new and simple peripheral blood biomarker Monocyte DIRECT LS-TA is proposed here which can be readily used in clinical setting. It can be used to differentiate bacterial infection and inform clinicians on the use of antibiotics. DIRECT LS-TA will emerge as a new kind of in vitro diagnostics (IVD) which can convey single cell-type gene expression information from PB samples. The new kind of IVD and uniqueness of the information, together with the ease of implementation will make it very useful in clinics.

## Materials and methods

### Datasets used in the analysis of gene expression of PB and monocytes

In order to identify monocyte informative genes that are suitable for the DIRECT LS-TA assay, the following gene expression datasets obtained from PB samples were used (Table 3). These datasets were available from the Gene Expression Omnibus (GEO), maintained by the US National Institutes of Health. Details were available under their accession numbers. The types of peripheral blood samples included whole blood (WB) and peripheral blood mononuclear cells (PBMCs). Specific cell types that have been further isolated and purified, such as isolated and purified monocytes, were also included in some datasets.

**Table 3:** List of PBMC or WB gene expression datasets used to identify monocyte informative genes.

Dataset accession number	Usage	Type of peripheral blood sample (WB or PBMC)	Methods used in data normalization	References
GSE138746 (bulk RNA sequencing)	1. To calculate the 90^th^ percentile value of the fold of expression in monocytes vs. PBMCs, and find out genes that meet the criteria for monocyte informative gene (See Figure of ICEBERG plot); 2. To evaluate the correlation between the biomarker of gene expression obtained by using DIRECT monocyte LS-TA and the gene expression of isolated monocytes (the gold standard)	PBMC and isolated and purified monocytes	Read count data normalised by EDASeq to align the upper quartile of each sample	[15]
GSE114407 (bulk RNA sequencing)	Ditto	PBMC and isolated and purified monocytes	Read count data normalised to transcript per million (TPM)	[16]
GSE60424 (bulk RNA sequencing)	Ditto	Whole blood and isolated and purified monocytes	Read count data normalised by EdgeR package	[17]
GSE107011 (bulk RNA sequencing)	To compare the gene expression of granulocytes and monocytes	PBMC and isolated and purified monocytes and granulocytes	Read count data normalised to transcript per million (TPM)	[6]

### Datasets used in the analysis of gene expression markers of monocytes for detection of bacterial infection

In order to identify monocyte marker that enables detection of bacterial infection, the following gene expression datasets obtained from peripheral blood samples were used (Table 4). GSE154918 is an RNA-sequencing dataset of PB of infection patients. It is used as the discovery dataset as RNA-sequencing results have better coverage of the transcriptome and are not restricted by the probe availability as in microarrays. After identifying potential monocyte DIRECT LS-TA RBBs that were activated in monocytes after bacterial infection, these RBBs were evaluated in 4 other replication datasets. They were also PB gene expression datasets but were analyzed on microarray platforms (Illumina and Affymetrix). Different gene expression platforms were included here to show that the new monocyte DIRECT LS-TA was a genuine phenomenon of monocytes and was not analysis platform-dependent. In all datasets, only results from patients with uncomplicated bacterial infection were used, while results from patients with sepsis (if any) were not included. Sepsis is a highly heterogeneous dysfunction of the immune system resulting from different etiologies and not only against bacterial infection. Quality control of samples in each dataset was applied as previously described which included detection of outliers by Mahalanobis distance metrics using a battery of conventional housekeeping genes [4].

**Table 4:** List of WB gene expression datasets used to identify monocyte DIRECT LS-TA biomarkers for differentiation of bacterial infection.

Dataset accession number	Grouping of samples and number of samples	Type of blood sample (WB or PBMC)	References
	Discovery Dataset		
GSE154918 (bulk RNA-seq)	Bacterial infection group: 11 Control group: 40 (samples from sepsis or follow-up patients were not included)	WB Read count data normalised to transcript per million (TPM)	[18]
	Replication Datasets		
GSE40012 (Illumina microarray)	Bacterial infection group: 30 (samples from patients in bacterial infection group on Day 1 and Day 2) Control group: 36 (samples on Day 1 and Day 5)	WB	[35]
GSE42026 (Illumina microarray)	Bacterial infection group: 18 Control group: 33	WB	[36]
GSE60244 (Illumina microarray)	Bacterial infection group: 22 Control group: 40	WB	[37]
GSE63990 (Affymetrix microarray)	Bacterial infection group: 71 Control group: 89	WB	[23]

### A mathematical framework for identification of monocyte informative genes with discernible expression in PB

**Figure 6:**
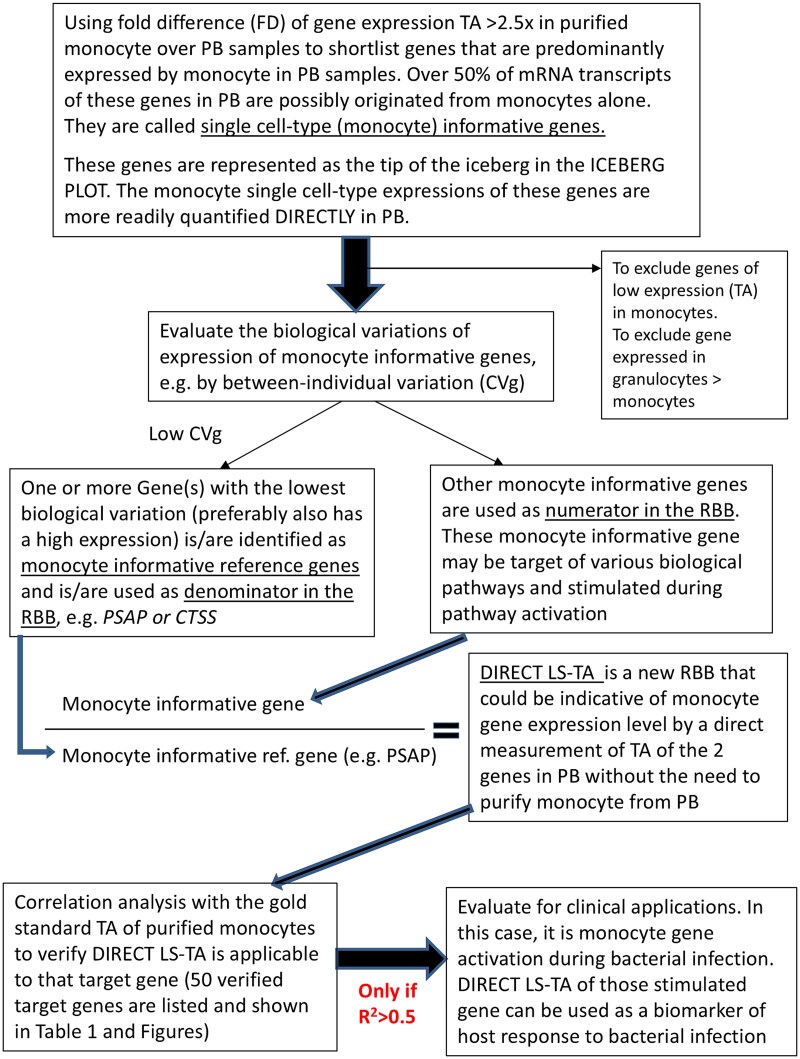
An overview of workflow to identify monocyte informative genes. This flow-chart explains the workflow for [1] shortlisting monocyte informative genes that can be directly quantified for monocyte single cell-type gene expression level from PB without sorting of monocytes, [2] identify monocyte informative reference genes to be used as the denominator of a new RBB called DIRECT LS-TA. DIRECT LS-TA of various target genes are validated as a good indicator of the target genes expression in purified monocytes by correlation analysis. And then they are further developed as reporter of host response to various stimulation or illness (e.g. bacterial infection).

An overview of the workflow to identify monocyte informative genes is shown in Fig. 6. Suppose a gene that is solely produced by monocytes in PB, its TA can be readily determined even in a cell-mixture sample of PB. These genes are typically used as cell-type marker genes, like CD14. However, genes with such an extreme degree of cell-type preferential expression are few. We develop a new concept of cell-type informative genes for a particular cell-type of interest which solely produces more than 50% of mRNA transcripts of the cell-type informative genes in a cell-mixture sample. With such a relaxed degree of preferential expression, a mathematical model was derived to define a new DIRECT LS-TA RBB of the ratio of a monocyte informative target gene to a monocyte informative reference gene, which will reflect the expression of the target gene in purified monocytes. Therefore, DIRECT LS-TA values of all shortlisted monocyte informative genes were derived from the bulk gene expression results in cell-mixture samples (e.g. WB) in datasets listed in Table 3 and their correlation with gold standard results (gene expression in purified monocytes) was analyzed. R^2^ of above 0.5 (r > 0.7) is used as a cutoff of acceptable correlation and these target genes are then selected for further evaluation of potential RBBs (Fig. 6).

**Figure 7:**
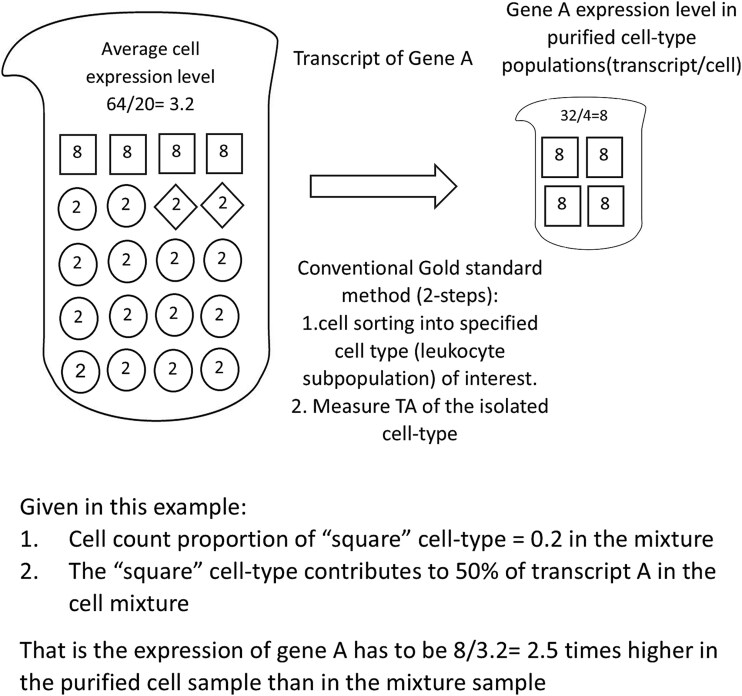
A schematic diagram of the concept for DIRECT LS-TA assay which can quantify expression of shortlisted monocyte informative genes directly in PB without requiring isolation of monocyte. The figure shows the original cell-mixture samples (e.g. PB) on the left and a component single cell-types (e.g. monocyte) sample isolated from the original cell-mixture sample by cell sorting. Monocyte, the cell-type of interest here, is shown as cells by square symbols. The proportional cell count of monocyte is set to 20% as in PBMC. Gene a has an average cellular expression level in an isolated monocyte sample that is above 2.5x (folds) higher than its average cellular expression level in a cell-mixture sample (for the square cell subpopulation, 8 (for isolated square cells)/3·2 (in cell-mixture sample) = 2·5 folds). 50% of gene a transcripts in the cell-mixture sample is contributed solely by monocyte (square symbol). The gene expression level is presented as transcript count per cell. For example, the cell-mixture sample has a total of 64 transcripts of gene a in 20 cells, the gene expression value = 64/20 = 3·2 transcript/cell. Such gene expression values are equivalent to relative expression quantification using housekeeping gene to normalize target gene expression. Genes with expression level 2·5x (folds) higher in the isolated square cell subpopulation than in the cell-mixture sample are candidate monocyte informative genes. In this example, to demonstrate the principle, it is assumed that the gene expression levels of other cells are known. In fact, we only need to know the expression levels of the isolated single cell-type sample of interest and the corresponding cell mixture sample to determine the required expression fold difference (FD) to shortlist single cell-type informative gene.

Similar to our previous publication [4], we defined cell-type informative genes as genes which are predominantly expressed by only a single cell-type (e.g. monocyte) to the extent that ≥50% of gene transcripts of these informative genes in a PB sample (for example, PBMCs) were contributed solely by that single cell-type [4]. It is shown schematically in Fig. 7 and in the supplementary material for the mathematical framework. Typically, the proportional cell count of monocytes in PBMCs was 10%–30% [38, 39]. By using the mathematical framework definition we described previously [4] and in the supplementary material, when the proportional cell count of monocyte P_(monocyte)_ was 20%, the expression of a monocyte informative gene in the purified monocyte sample needed to be 2.5 times higher (eq’n 6 in supplementary material) than that in the cell-mixture sample, as illustrated in Fig. 7. The monocyte informative genes in the cell-mixture blood sample were identified by using these conditions. Expression data from the isolated monocyte sample and cell-mixture sample (PBMCs or WB) in datasets from GEO (Table 3) were used to determine which genes were monocyte informative genes. The results are also shown by ICEBERG plots in Fig. 1.

To develop the DIRECT LS-TA RBB, one or more denominator genes are required. They are selected among the 50 shortlisted monocyte informative genes. The denominator gene (monocyte informative reference gene) is a monocyte informative gene with the lowest biological variation. Therefore, the coefficient of variation (CV) was calculated for each monocyte informative genes to find those with the lowest CV. As shown in the ICEBERG plots in Fig. 1, conventional housekeeping genes (e.g. *ACTB, UBC* and *GAPDH*) cannot be used here as they are all below the required X50 threshold, as expressed across all cell-types in PB but not specific to monocytes.

### To derive the new monocyte DIRECT LS-TA results from bulk transcriptome data of PB samples


\begin{align*} \mathrm{Monocyte}\ \mathrm{DIRECT}\ \mathrm{LS}-\mathrm{TA}=\mathrm{monocyte}\ \mathrm{informative}\\{\mathrm{gene}}_{\left(\mathrm{WB}\right)}/\mathrm{monocyte}\ \mathrm{reference}\ {\mathrm{gene}}_{\left(\mathrm{WB}\right)} \end{align*}

That is, the new RBB is a ratio of TA of 2 genes in the cell-mixture sample (e.g. WB or PBMC).

In the gene expression dataset, the gene expression values are log transformed. Therefore, this RBB can also take its log form as follows:


\begin{align*}& \mathrm{Log}\ \left(\mathrm{monocyte}\ \mathrm{DIRECT}\ \mathrm{LS}-\mathrm{TA}\right)\\&=\mathrm{Log}\ \left(\mathrm{monocyte}\ \mathrm{informative}\ {\mathrm{gene}}_{\left(\mathrm{WB}\right)}\right)\\&-\mathrm{Log}\ \left(\mathrm{monocyte}\ \mathrm{reference}\ {\mathrm{gene}}_{\left(\mathrm{WB}\right)}\right) \end{align*}

Conceptually, this is similar to the results of delta CT (ΔCT) in quantitative PCR (qPCR) relative quantification experiments. The difference of threshold cycles (CT) of the target gene and the normalisation reference gene (typically one or more housekeeping genes) is called delta CT (ΔCT) in qPCR relative quantification. In order to understand the degree of activation after stimulation or having a disease, such delta CT value is compared to the delta CT of a control individual (or a calibrator sample), this new result is called delta–delta CT (ΔΔCT).

To reflect the extent of gene activation compared to the controls, a result that is conceptually similar to delta–delta CT (ΔΔCT) can be obtained for DIRECT LS-TA RBB. The median DIRECT LS-TA result of the control group is used as the calibrator sample, and it is subtracted from DIRECT LS-TA results of all subjects. In other words, the median DIRECT LS-TA value of the control group was set to zero, and all other DIRECT LS-TA results were standardised against this median. In statistical terms, it is called multiples of median (MoM). The MoM results will be comparable to ΔΔCT results when DIRECT LS-TA is used in prospective patients with qPCR or dPCR assays.

Since experiments performed on different analytical platforms yield results in different units, MoM is a method of choice to standardise results obtained from different assays. For example, it is commonly used in prenatal biochemical screening [40], and in cytokine assays [41].

### Data analysis and statistics methods used to evaluate performance of DIRECT LS-TA RBB to detect the host response to bacterial infection

In the discovery dataset, GSE154918, monocyte DIRECT LS-TA results of 50 monocyte informative genes (see Table 1) were calculated. And the difference of monocyte DIRECT LS-TA result of each target gene was compared between the control group (n = 40) and the patient group with uncomplicated bacterial infection (n = 11). A non-parametric statistical (Wilcoxon–Mann–Whitney test) test was used to analyse the group difference. As 50 target genes and 2 reference genes would result in performing 100 statistical tests, a multiple testing correction by the Bonferroni method was used, and the type I error is set to 1 × 10^−5^ (equivalent to corrected *P* < 0.001).

The seven best discriminating monocyte DIRECT LS-TA biomarkers were then evaluated in the replication samples for group-wise difference and area-under-curve (AUC) in receiver-operating characteristic (ROC) analysis.

## Supplementary Material

HMG-2025-OA-00361_Supplementary_material_and_figure_ddaf103

HMG-2025-OA-00361_Supplementary_material-Effect_PSAP_and_CTSS_ddaf103
