# Supplementary material for: Direct single cell-type gene expression analysis in peripheral blood: novel ratio-based gene expression biomarkers using 2 novel monocyte reference genes (PSAP and CTSS) for detection of bacterial infection
Source: Hum Mol Genet. 2025 Jun 23;34(17):1458–70. doi: 10.1093/hmg/ddaf103 (PMC12368773; doi:10.1093/hmg/ddaf103)
Supplement: HMG-2025-OA-00361_Supplementary_material_and_figure_ddaf103 [file hmg-2025-oa-00361_supplementary_material_and_figure_ddaf103.pdf]

Supplementary material

Mathematical framework to evaluate FD required for single cell-type informative genes

This is a general mathematic model to described the percentage contribution of TA (*C%*) by a single cell-type $_1$ (e.g. monocyte) in a cell-mixture sample (e.g. PB). The specified single-cell-type is monocyte, therefore, $TA_{(1)}$ is the expression level of purified monocyte which has a proportional cell count of $P_{(1)}$ in PB. $P_{(1)}$,proportional cell count of monocyte is assigned a values of 0.2 for monocytes in PBMC. The summation in the right side of the equation provides a generalised model for different type of cell-mixture samples composed of N different cell-types.

$$TA_{(1)} \cdot P_{(1)} = \frac{C\%}{(1-C\%)} \sum_{i=2}^{N} TA_{(i)} \cdot P_{(i)} \qquad \text{(eq'n 1)}$$

The generalised formula is difficult to apply as expression levels $TA_{(i)}$ and proportional cell-count $P_{(i)}$ of all cell-types present in the samples are required which are not available or not feasible to perform cell sorting of one cell-type without loss of another cell-type.

A more practical scenario of experimental setting is that TA of the single cell-type of interest and TA of the cell-mixture are quantified. Therefore, in the present context, researchers quantified the gene expression of the PBMC samples and purified monocyte from the same individual. So we have measured results for TA(monocyte) and TA(PBMC). The task is to understand how much fold difference (FD) which is the ratio of TA(monocyte) to TA(PBMC), is required to satisfy C% ≥ 50% (i.e. The majority of transcript in the PBMC are originated from monocytes).

Let *X* = FD at a particular C%, therefore

$$X = \frac{TA_{(1)}}{TA_{(cell\ mixture)}} \qquad \text{(eq'n 2)}$$

A simplified model of 2 cell-types (specified cell-type 1 and other) is used to show the proof which is also valid for mixture samples of multiple cell-types.

$$TA_{(cell\ mixture)} = TA_{(1)} \cdot P_{(1)} + TA_{(other)} \cdot (1 - P_{(1)}) \qquad \text{(eq'n 3)}$$

For the DIRECT LS-TA assay specification of C% ≥ 50%, the required FD is called *X50.*

When C% = 50%

$$TA_{(1)} \cdot P_{(1)} = TA_{(other)} \cdot (1 - P_{(1)}) \qquad \text{(eq'n 4)}$$

Therefore

$$TA_{(0ther)} = \frac{TA_{(1)} \cdot P_{(1)}}{(1 - P_{(1)})} \qquad \text{(eq'n 5)}$$

Then, substitute into eq'n 2

$$X50 = \frac{TA_{(1)}}{TA_{(1)} \cdot P_{(1)} + \frac{TA_{(1)} \cdot P_{(1)}}{(1 - P_{(1)})} \cdot (1 - P_{(1)})} \qquad \text{(eq'n 6)}$$

Therefore

$$X50 = \frac{1}{2 \cdot P_{(1)}} \qquad \text{(eq'n 7)}$$

X50 for monocyte with a proportional cell count 0.2 = 2.5x

In summary, FD of expression in purified monocytes over PBMC exceeding 2.5 time is an indication that these genes are predominantly produced by monocyte in PB and their monocyte expression levels could be readily quantified directly from PBMC. (Tip of the ICEBERG)

Supplementary Figure S1

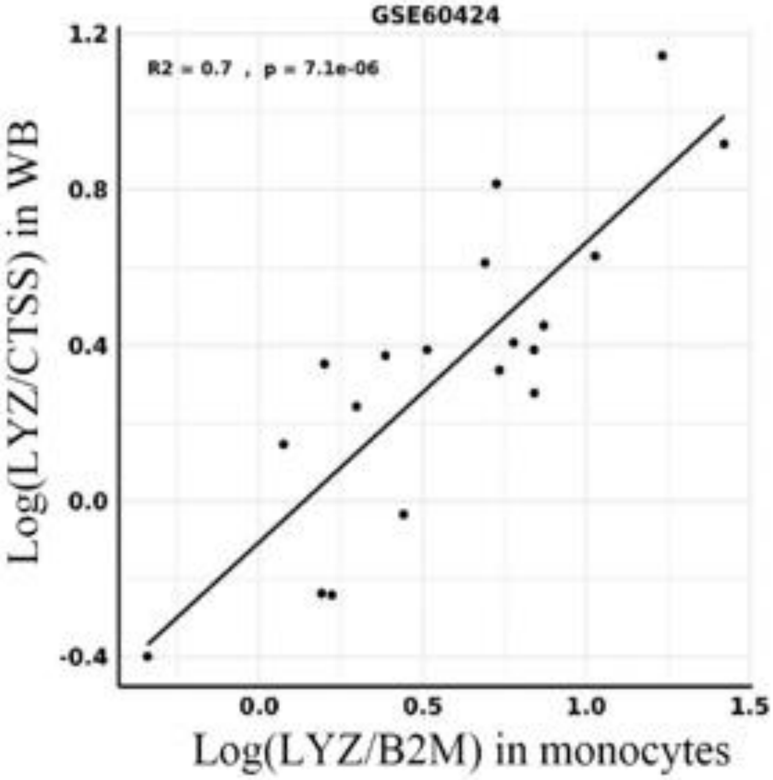

<u>Correlation between the results of biomarkers obtained by the monocyte DIRECT LS-TA assay and the expression of target gene *LYZ* detected in isolated monocytes by the traditional method in the GSE60424 dataset</u>. *LYZ* which is highly expressed genes in monocytes, are selected as the target genes, *CTSS* identified in the present study is used as reference gene in the cell-mixture sample. The X-axis shows the determination of gene expression of *LYZ* in isolated and purified monocytes, using *B2M* as a conventional housekeeping gene, i.e. Log ($LYZ_{\text{(monocytes)}}$ /$B2M_{\text{(monocytes)}}$) , where the X-axis here is the gold standard. The Y-axis monocyte DIRECT LS-TA in WB, wherein *CTSS* is used as reference gene, i.e. Log ($LYZ_{\text{(WB)}}$ / $CTSS_{\text{(WB)}}$). The correlation was high between monocyte DIRECT LS-TA of *LYZ* in WB with monocyte *LYZ* expression (R^2=0.7).

Supplementary Figure S2

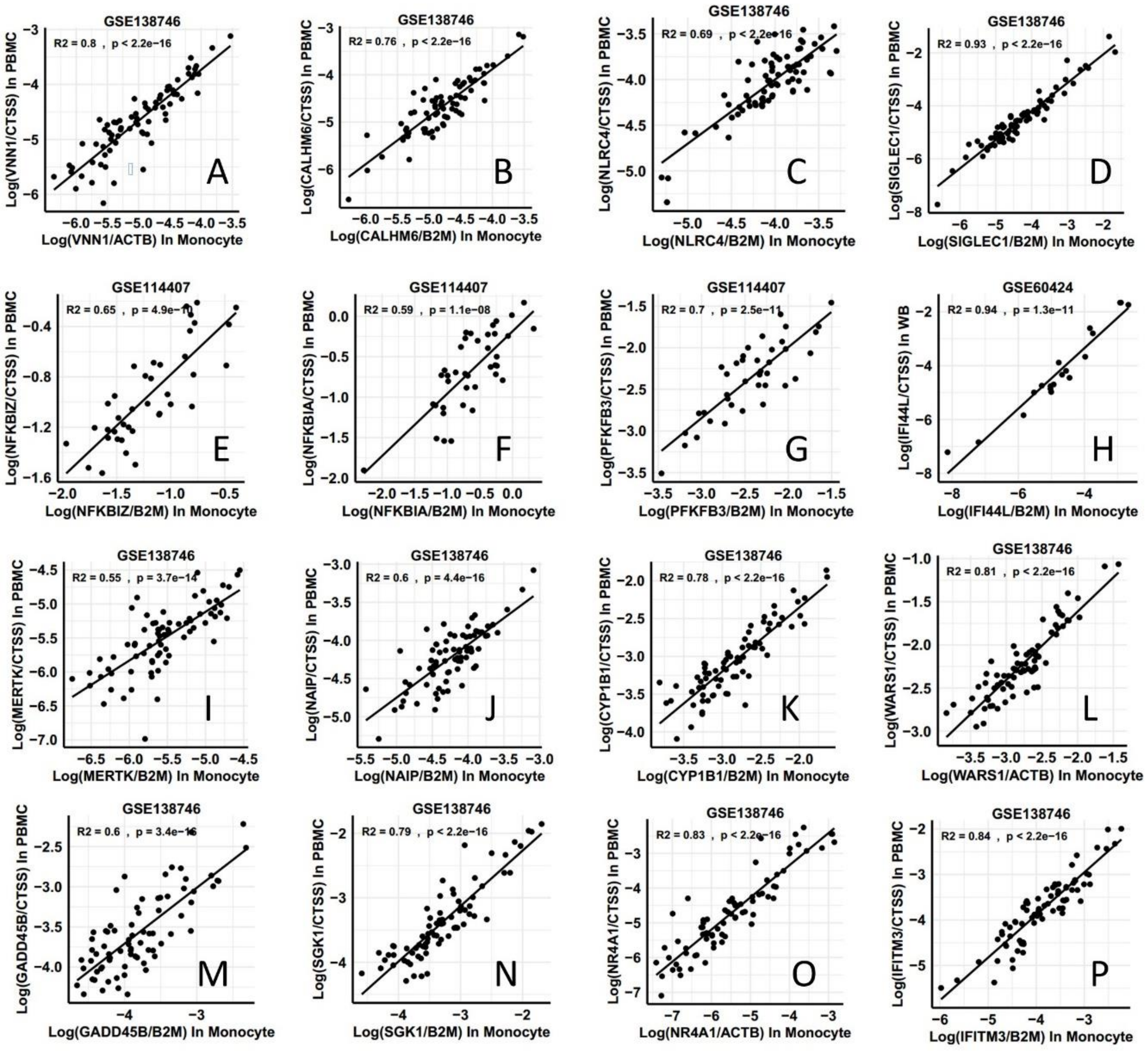

The correlation between monocyte DIRECT LS-TA for monocyte informative target genes measured in PB samples and expression levels of the same target genes in isolated monocytes obtained by the traditional method, using *CTSS* as a monocyte informative reference gene. Figure S2A shows the *VNN1* gene expression in monocytes determined by the method of DIRECT LS-TA and the traditional method. The Y-axis is the ratio of Log ($\mathit{VNN1}_{\text{(PBMC)}}$ / $\mathit{CTSS}_{\text{(PBMC)}}$) determined directly from PB samples (i.e., monocyte DIRECT LS-TA biomarker of *VNN1* gene). The X-axis is the gold standard, using the traditional method to detect *VNN1* expression after purification of monocytes, and a conventional housekeeping gene (*B2M*) for normalization, i.e. Log ($\mathit{VNN1}_{\text{(monocytes)}}$ /$\mathit{B2M}_{\text{(monocytes)}}$). As shown in Figure S2A, there is a good correlation between the two. Evaluation of the

performance of other monocyte informative genes using DIRECT LS-TA in peripheral blood is shown in Figure S2B-S2P, where the genes are *CALHM6, NLRC4, SIGLEC1, NFKBIZ, NFKBIA, PFKFB3, IFI44L, MERTK, NAIP, CYP1B1, WARS1, GADD45B, SGK1, NR4A1* and *IFITM3* respectively. Dataset accession numbers for data sources are shown above Figures S2A-S2P. All monocyte informative genes show a high and statistically significant correlation ($R^2 > 0.5$).

Supplementary Figure S3

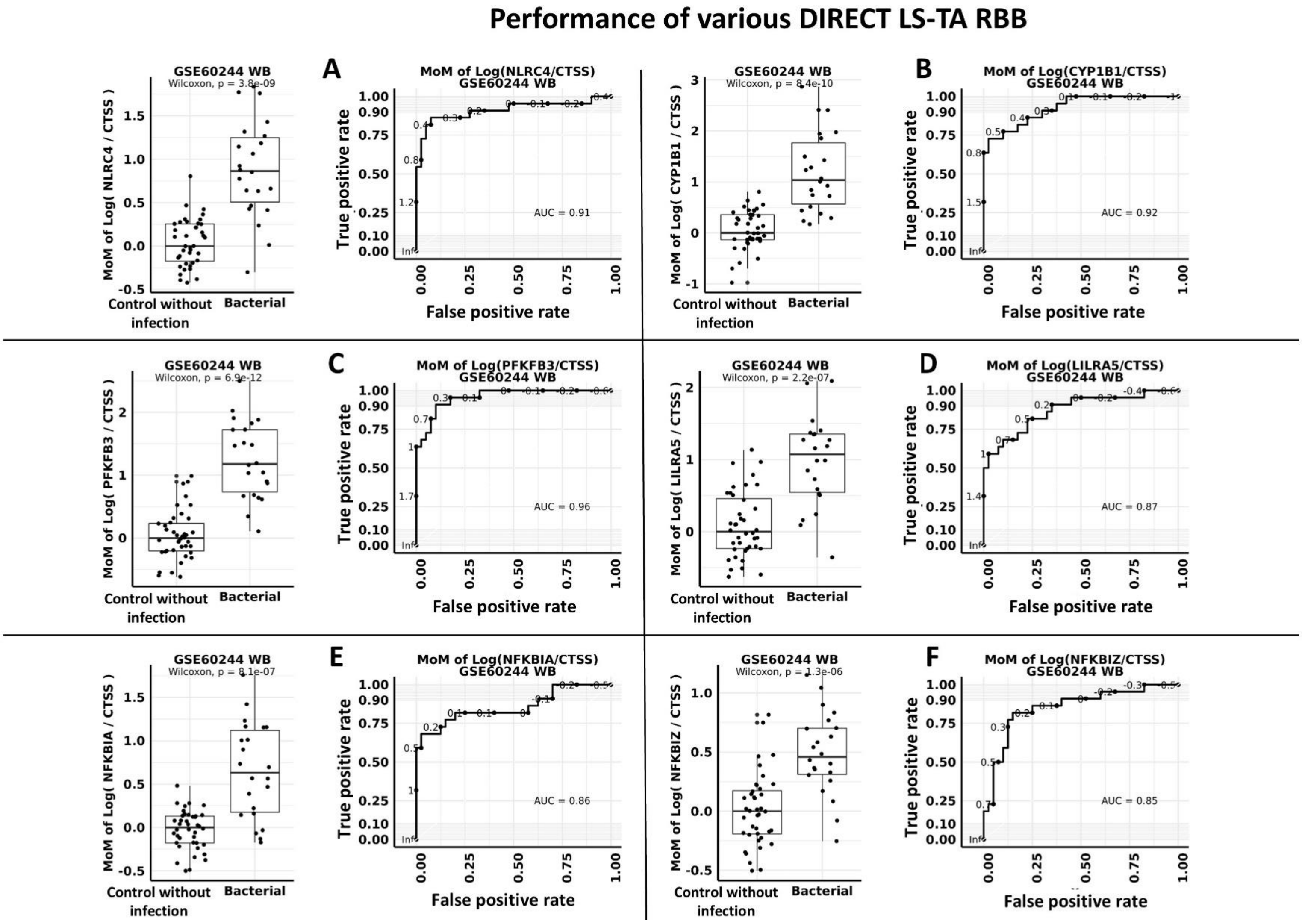

<u>Analysis of Monocyte DIRECT LS-TA assays for six additional target gene using *CTSS* as reference gene (i.e., *NLRC4*, *CYP1B1*, *PFKFB3*, *LILRA5*, *NFKBIA*, and *NFKBIZ*, respectively) and their receiver operating characteristic (ROC) curve analysis of the discriminative performance in differentiating uncomplicated bacterial infection</u>. Supplementary Figure S3A-S3F show the diagnostic performance of six additional target genes of monocytes, the expression of which are activated after bacterial infection. Here, the monocyte DIRECT LS-TA is calculated using *CTSS* as a reference gene. For each gene, the difference between MoM of Monocyte DIRECT LS-TA in peripheral blood between the control group and the bacterial infection group is shown by boxplots (left). The right panel shows results of ROC analysis.
