# Supplementary material for: Direct single cell-type gene expression analysis in peripheral blood: novel ratio-based gene expression biomarkers using 2 novel monocyte reference genes (PSAP and CTSS) for detection of bacterial infection
Source: Hum Mol Genet. 2025 Jun 23;34(17):1458–70. doi: 10.1093/hmg/ddaf103 (PMC12368773; doi:10.1093/hmg/ddaf103)
Supplement: HMG-2025-OA-00361_Supplementary_material-Effect_PSAP_and_CTSS_ddaf103 [file hmg-2025-oa-00361_supplementary_material-effect_psap_and_ctss_ddaf103.pdf]

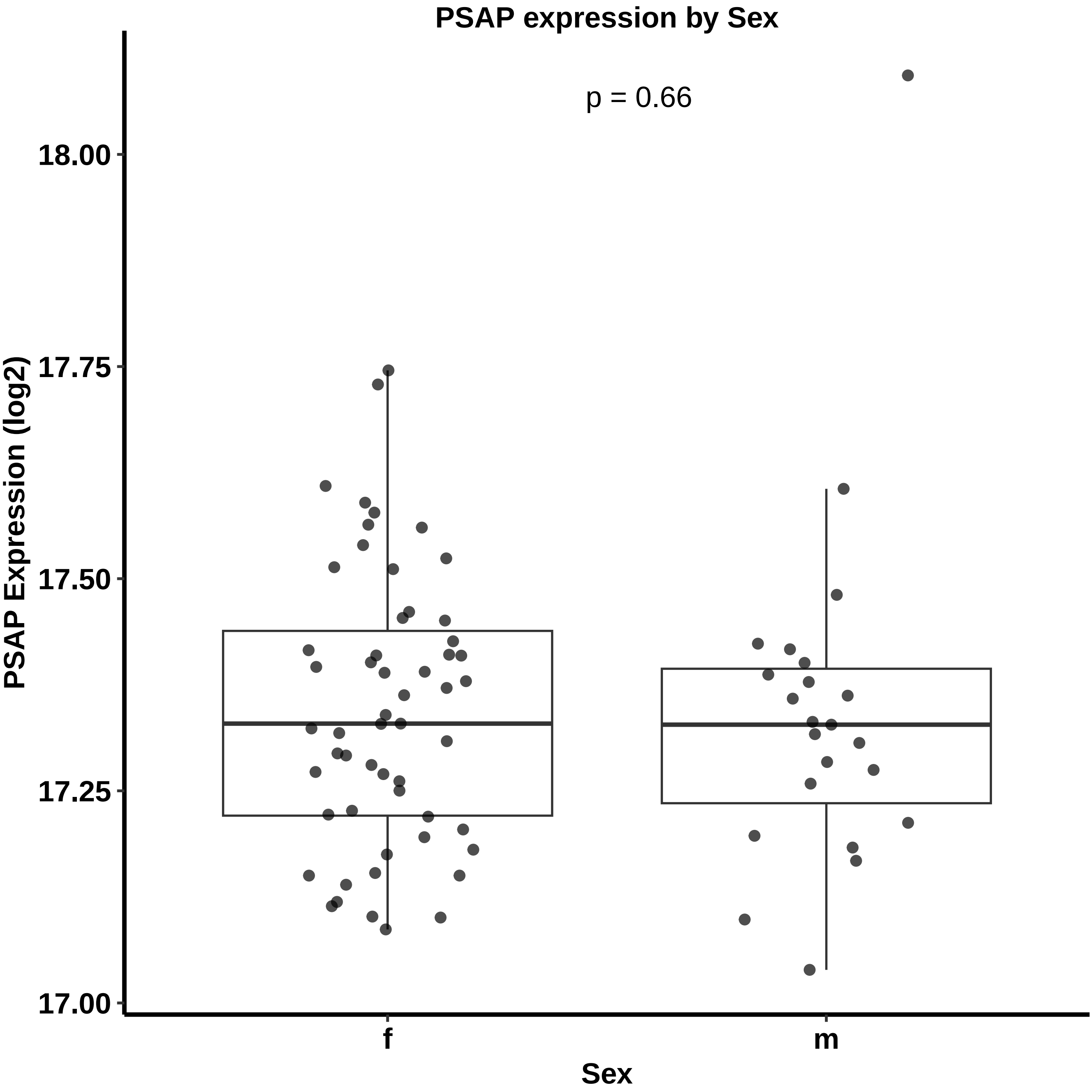

PSAP expression by Sex
p = 0.66
18.00
17.75
17.50
17.25
17.00
PSAP Expression (log2)
f
m
Sex

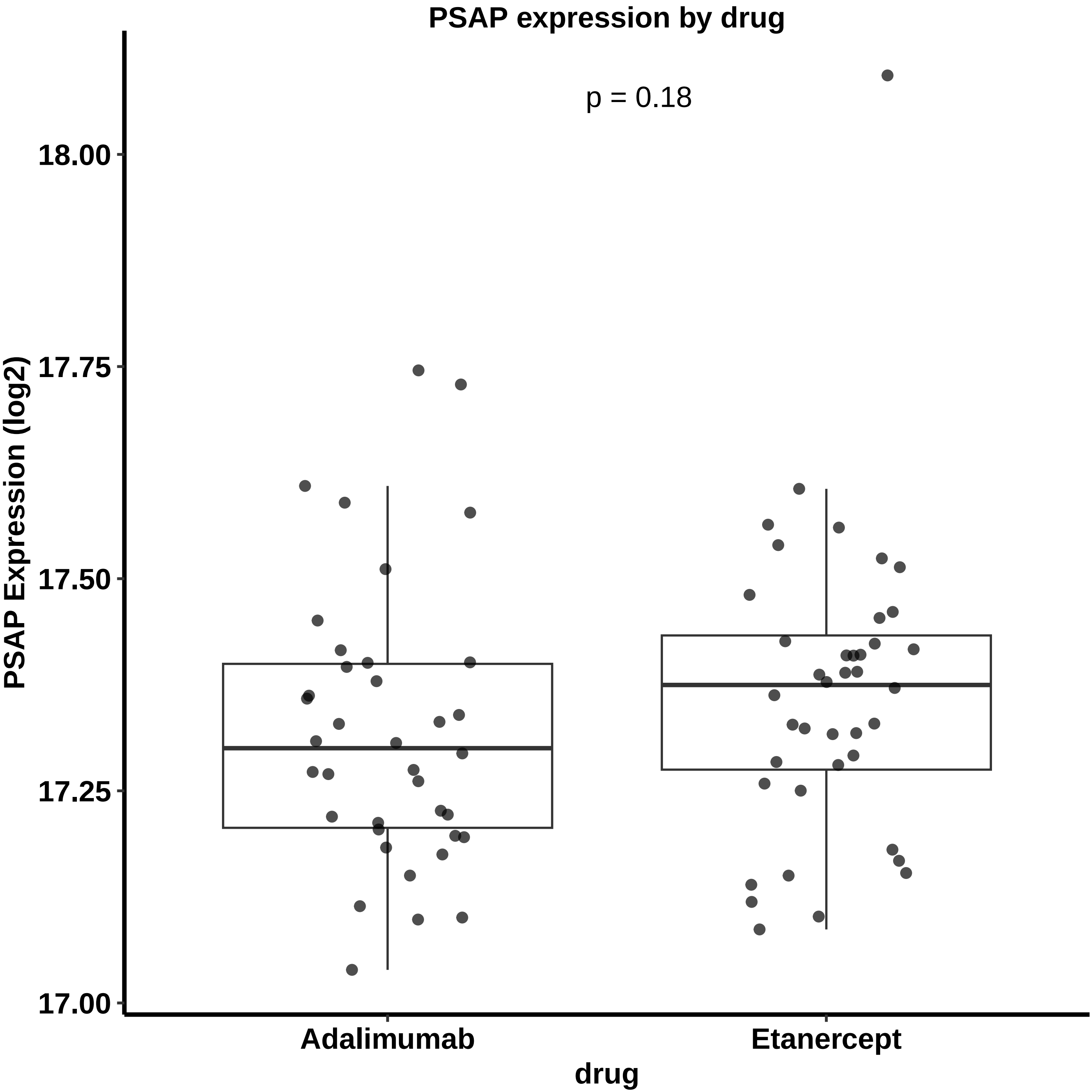
PSAP expression by drug
p = 0.18
PSAP Expression (log2)
18.00
17.75
17.50
17.25
17.00
Adalimumab
Etanercept
drug

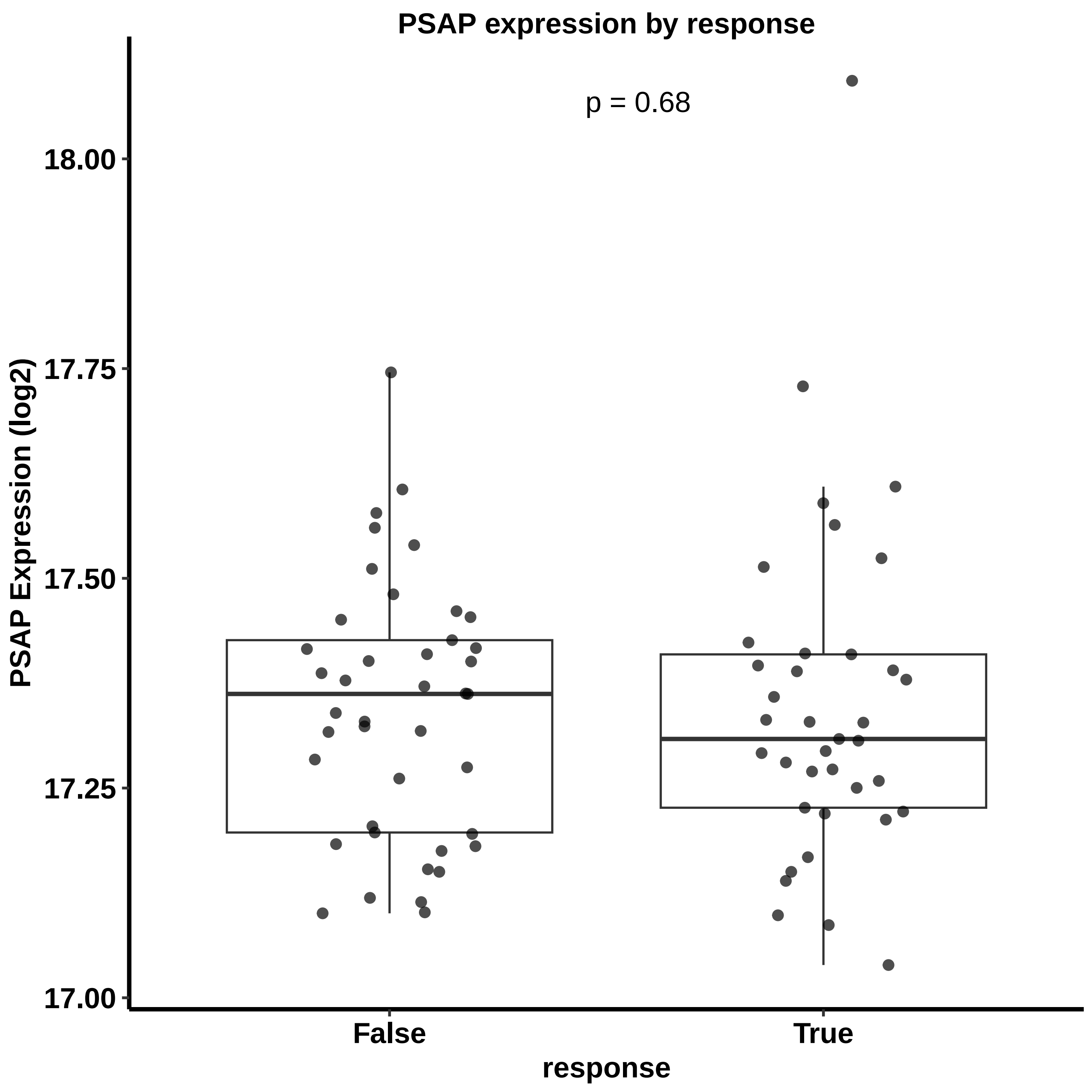

PSAP expression by response
p = 0.68
PSAP Expression (log2)
18.00
17.75
17.50
17.25
17.00
False
True
response

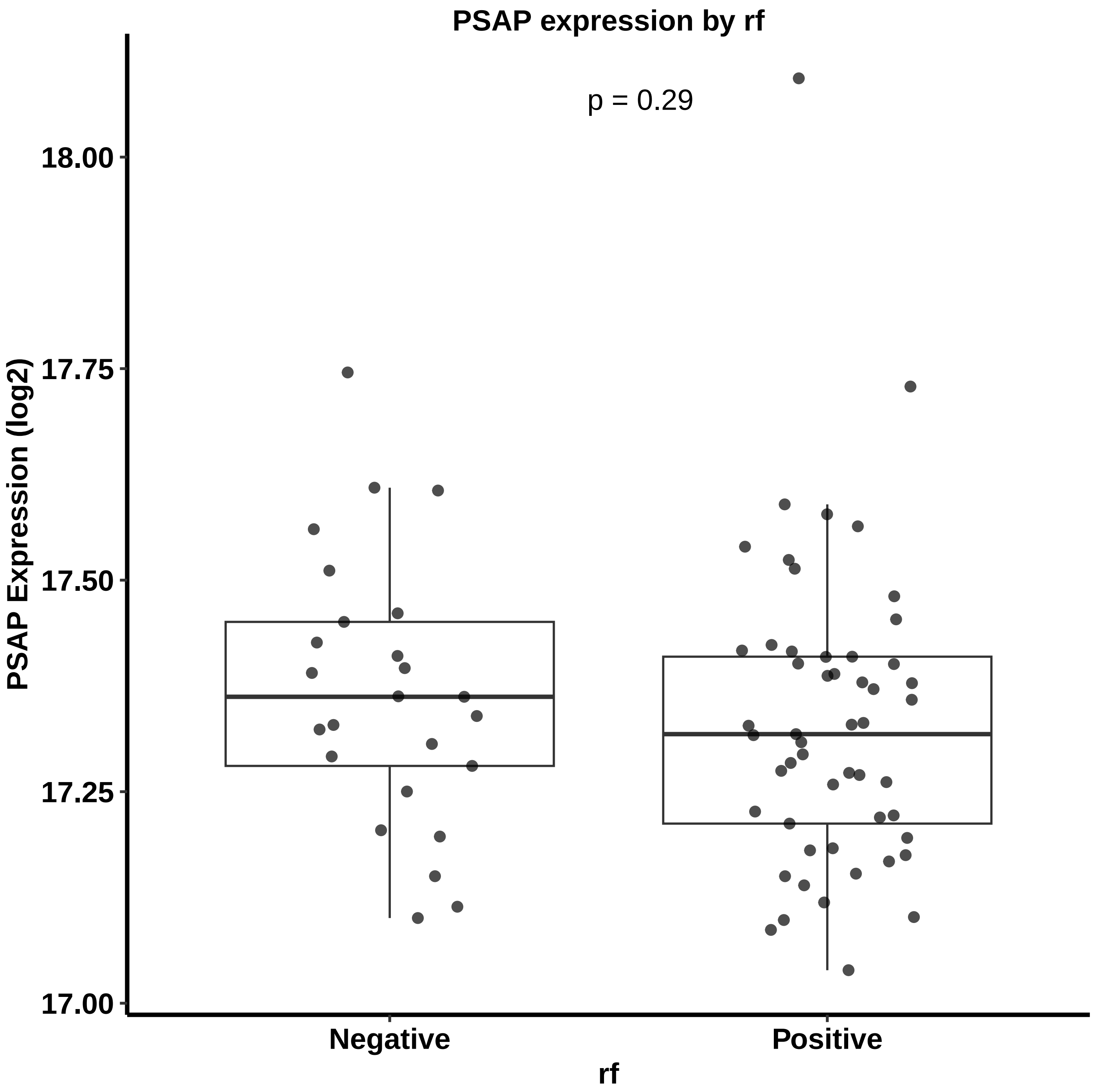
PSAP expression by rf
p = 0.29
PSAP Expression (log2)
18.00
17.75
17.50
17.25
17.00
Negative
Positive
rf

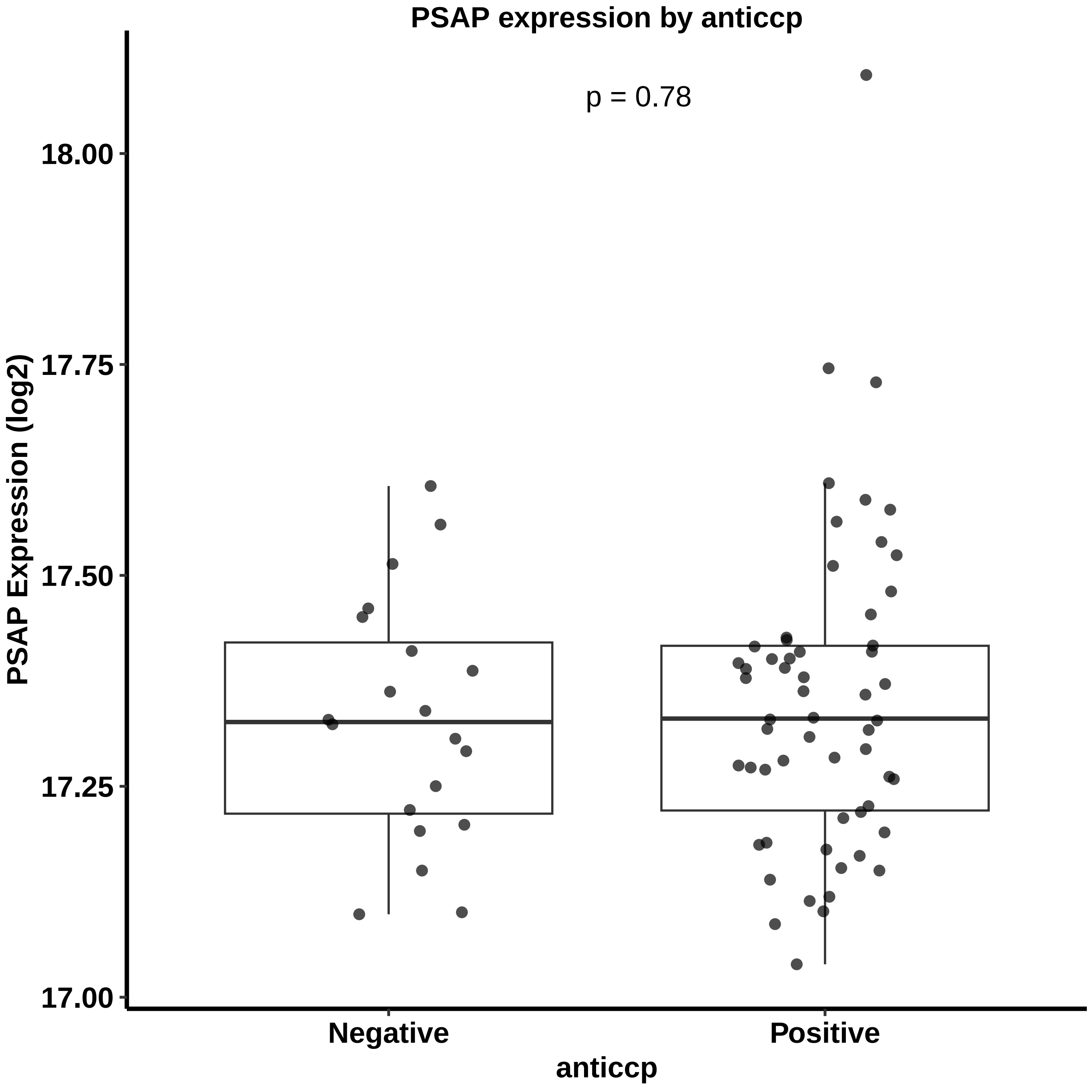
PSAP expression by anticcp
p = 0.78
PSAP Expression (log2)
18.00
17.75
17.50
17.25
17.00
Negative
Positive
anticcp

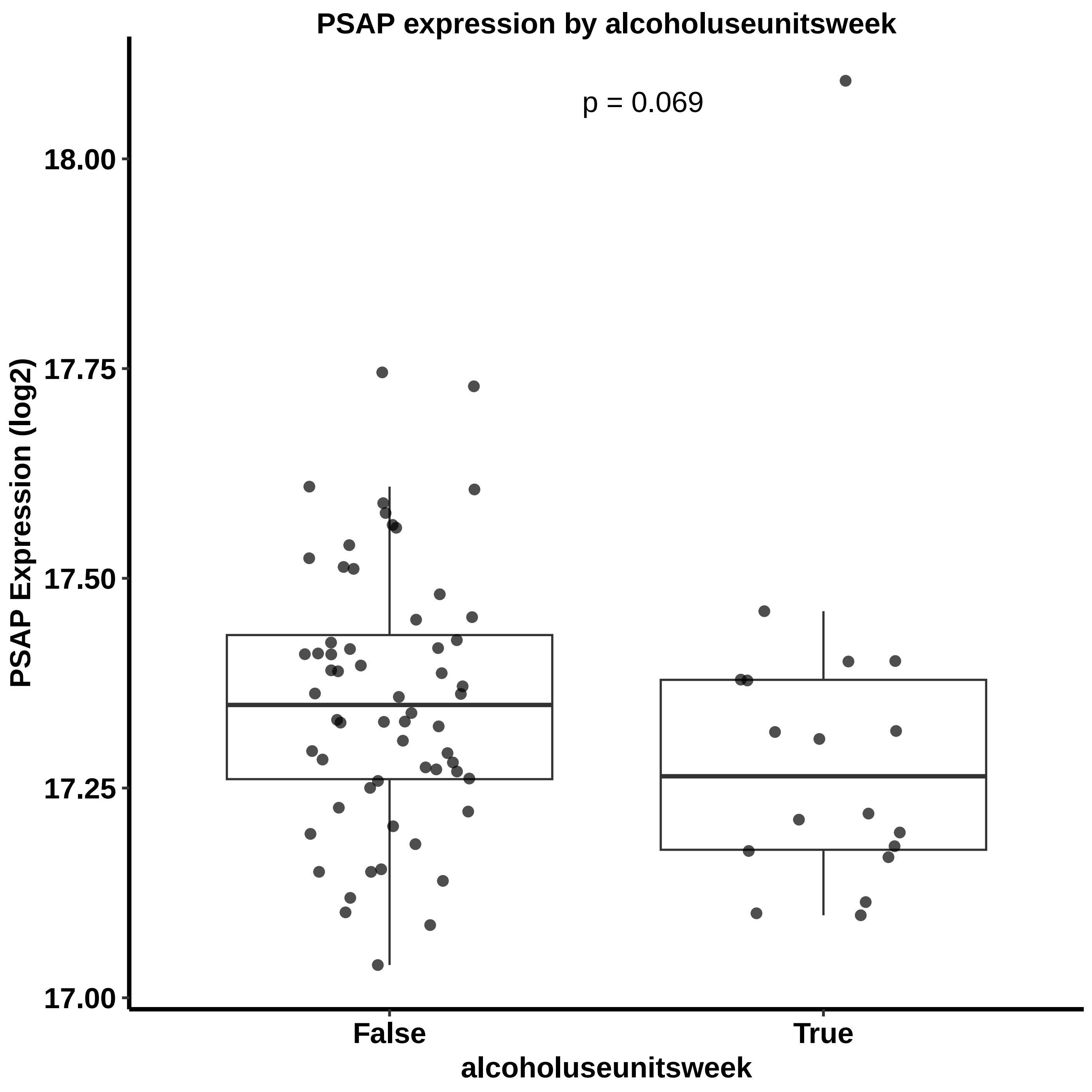
PSAP expression by alcoholuseunitsweek
p = 0.069
PSAP Expression (log2)
18.00
17.75
17.50
17.25
17.00
False
True
alcoholuseunitsweek

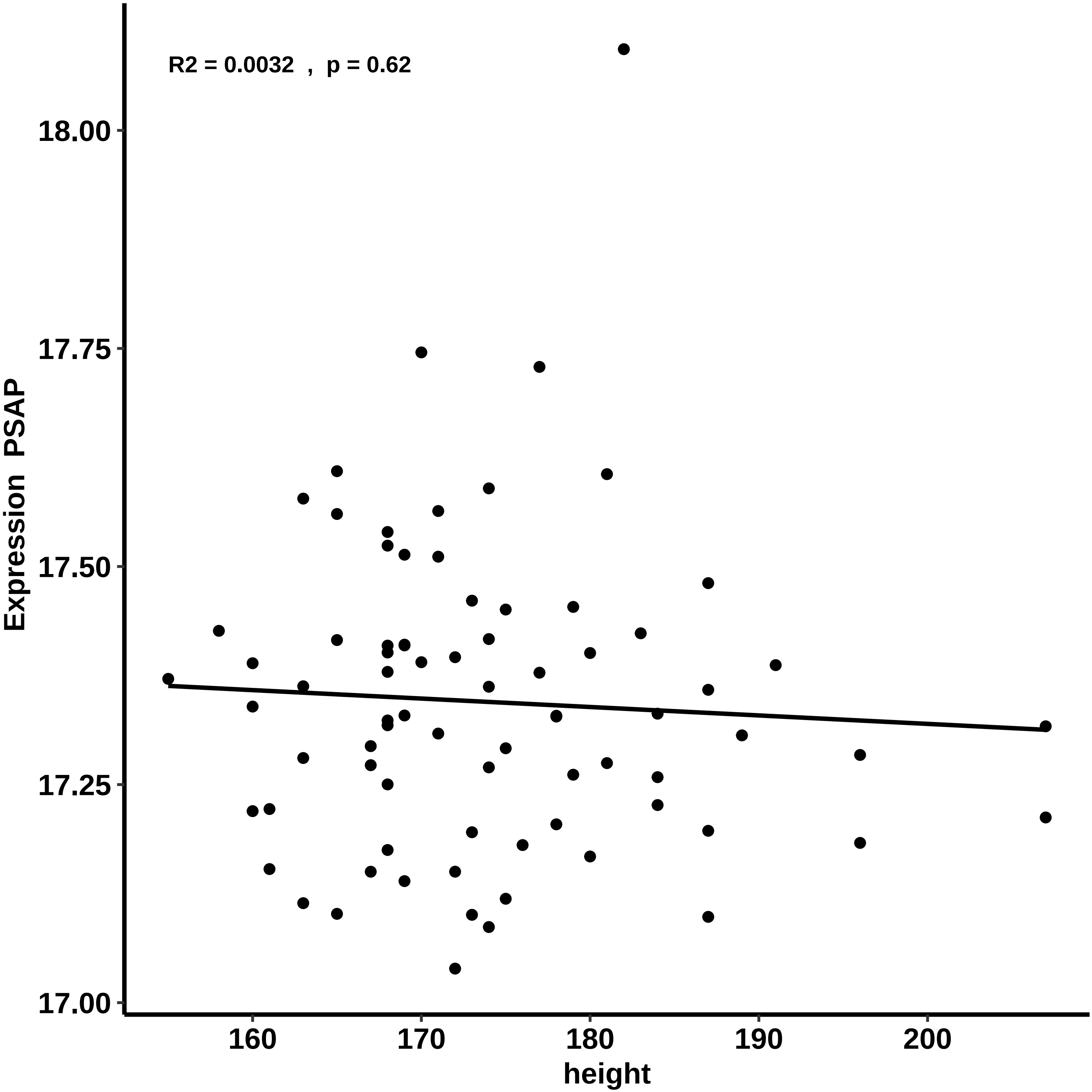
R2 = 0.0032 , p = 0.62
18.00
17.75
17.50
17.25
17.00
Expression PSAP
160
170
180
190
200
height

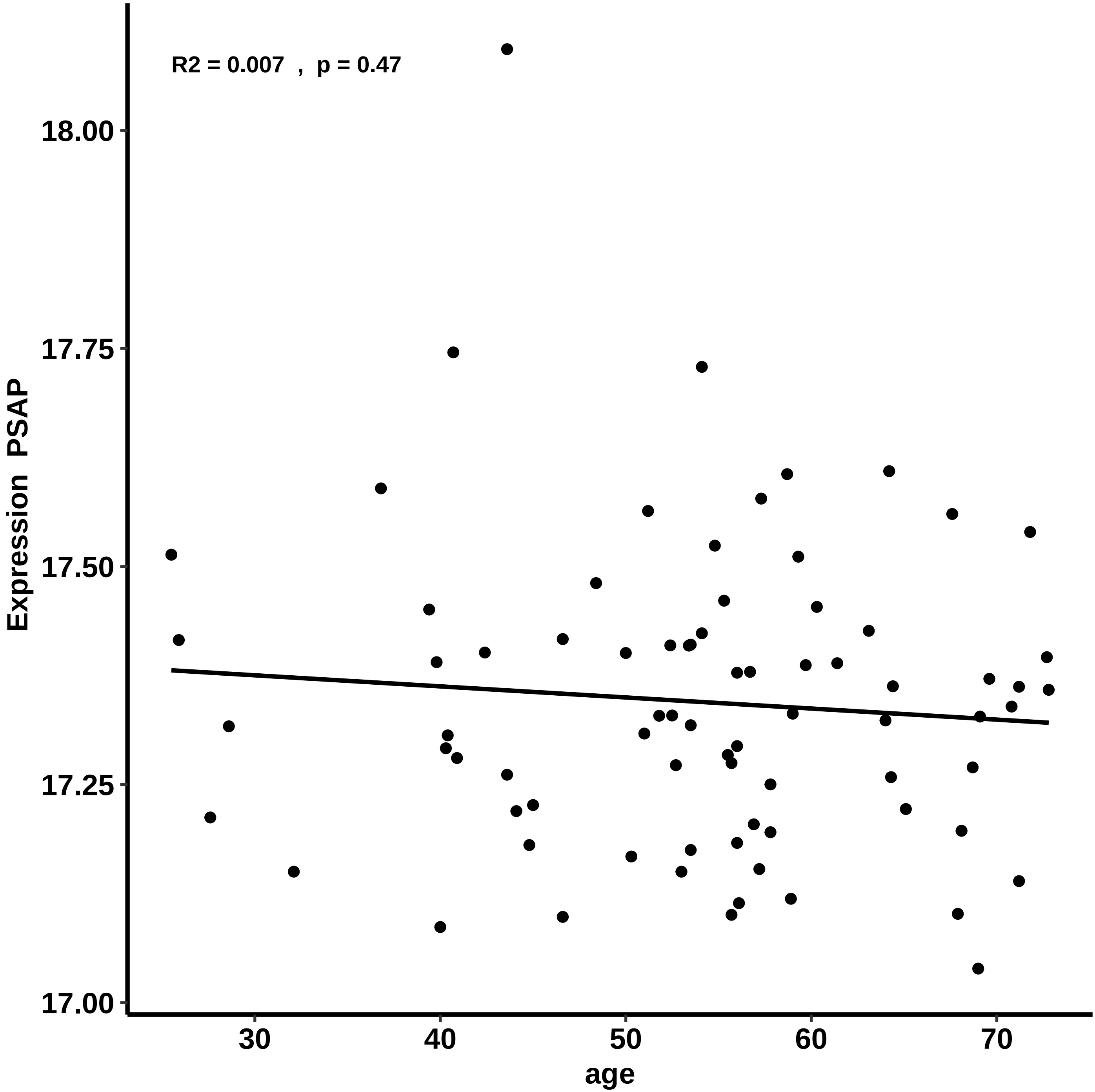
R2 = 0.007 , p = 0.47
Expression PSAP
18.00
17.75
17.50
17.25
17.00
30
40
50
60
70
age

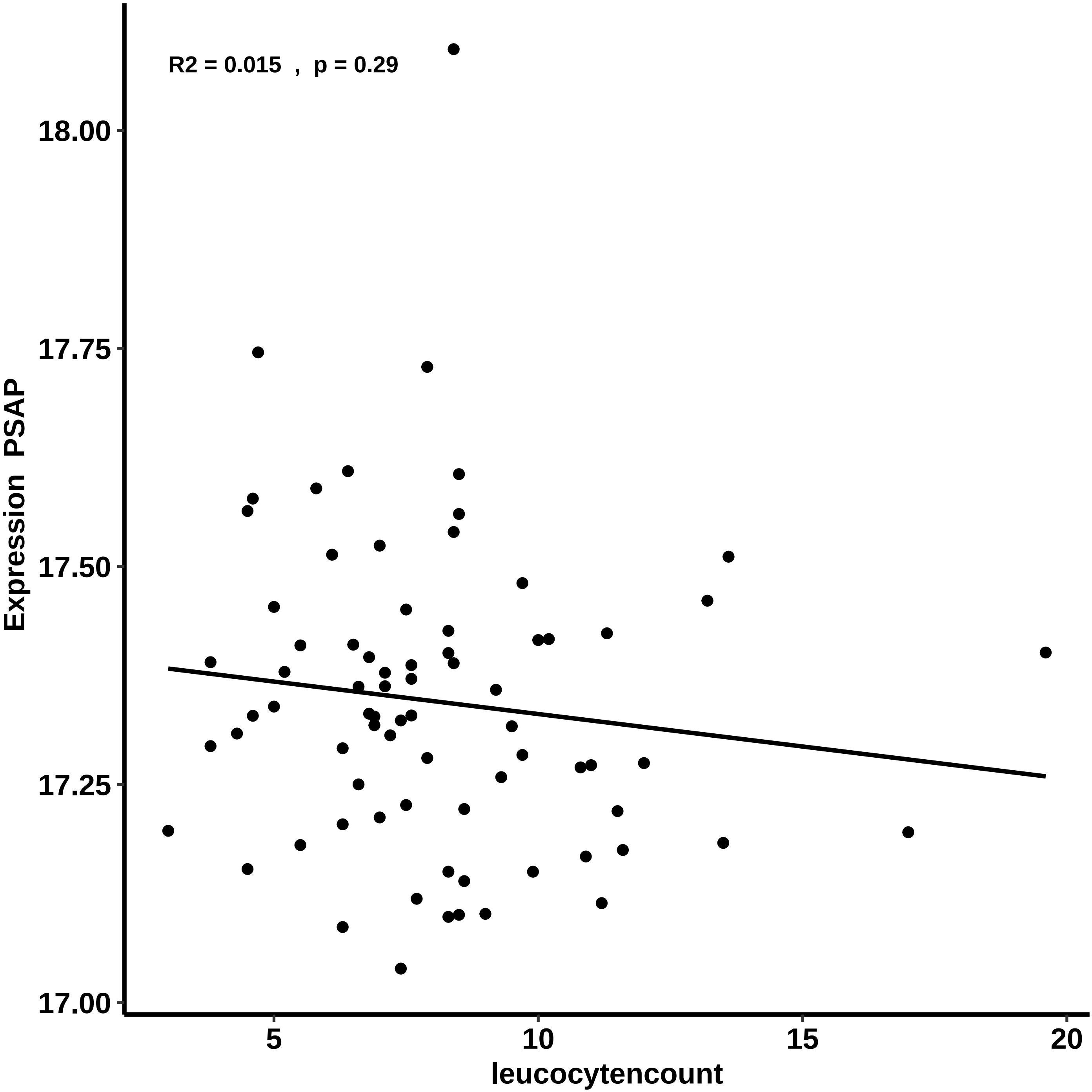
R2 = 0.015 , p = 0.29
18.00
17.75
17.50
17.25
17.00
Expression PSAP
5
10
15
20
leucocytencount

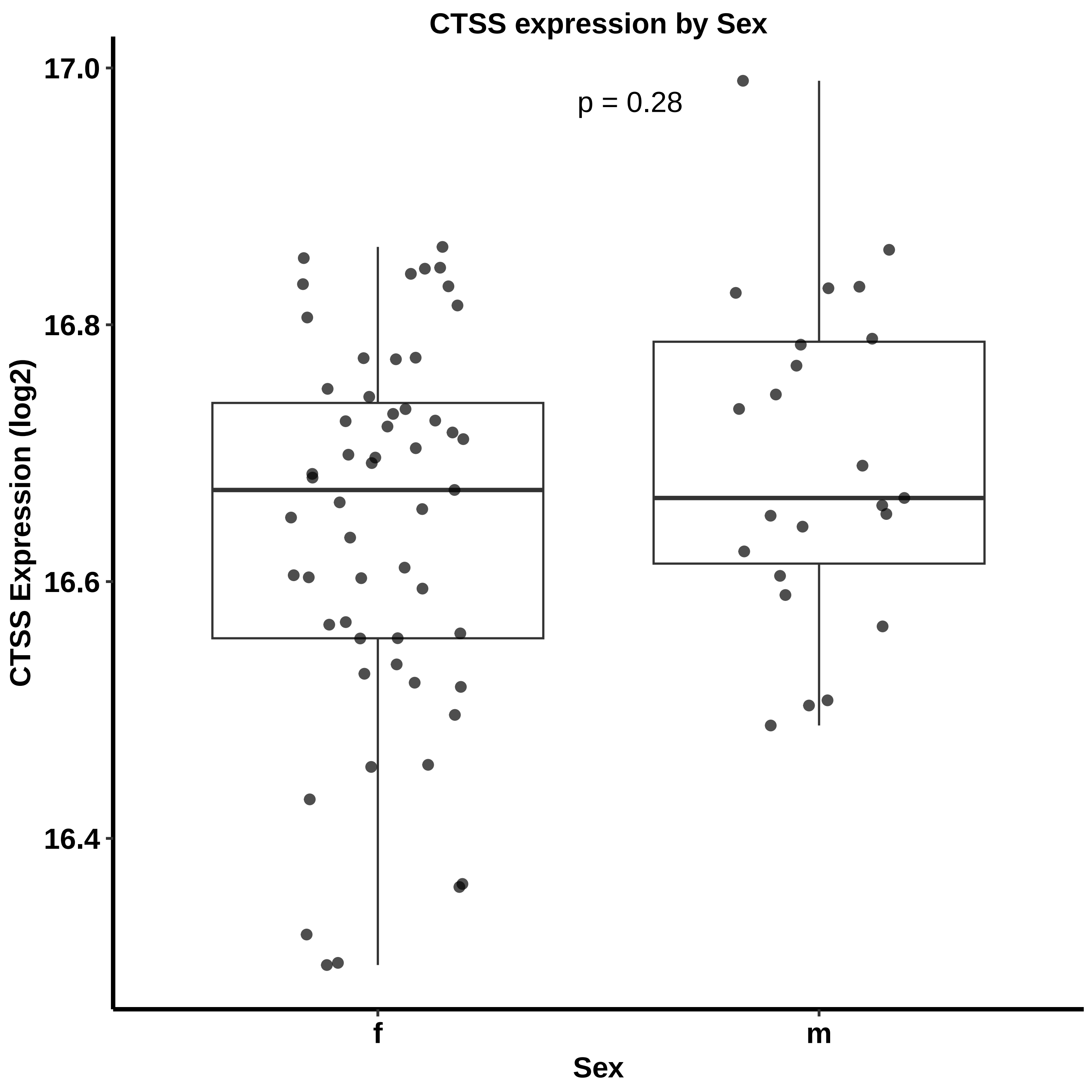
CTSS expression by Sex
p = 0.28
17.0
16.8
16.6
16.4
CTSS Expression (log2)
f
m
Sex

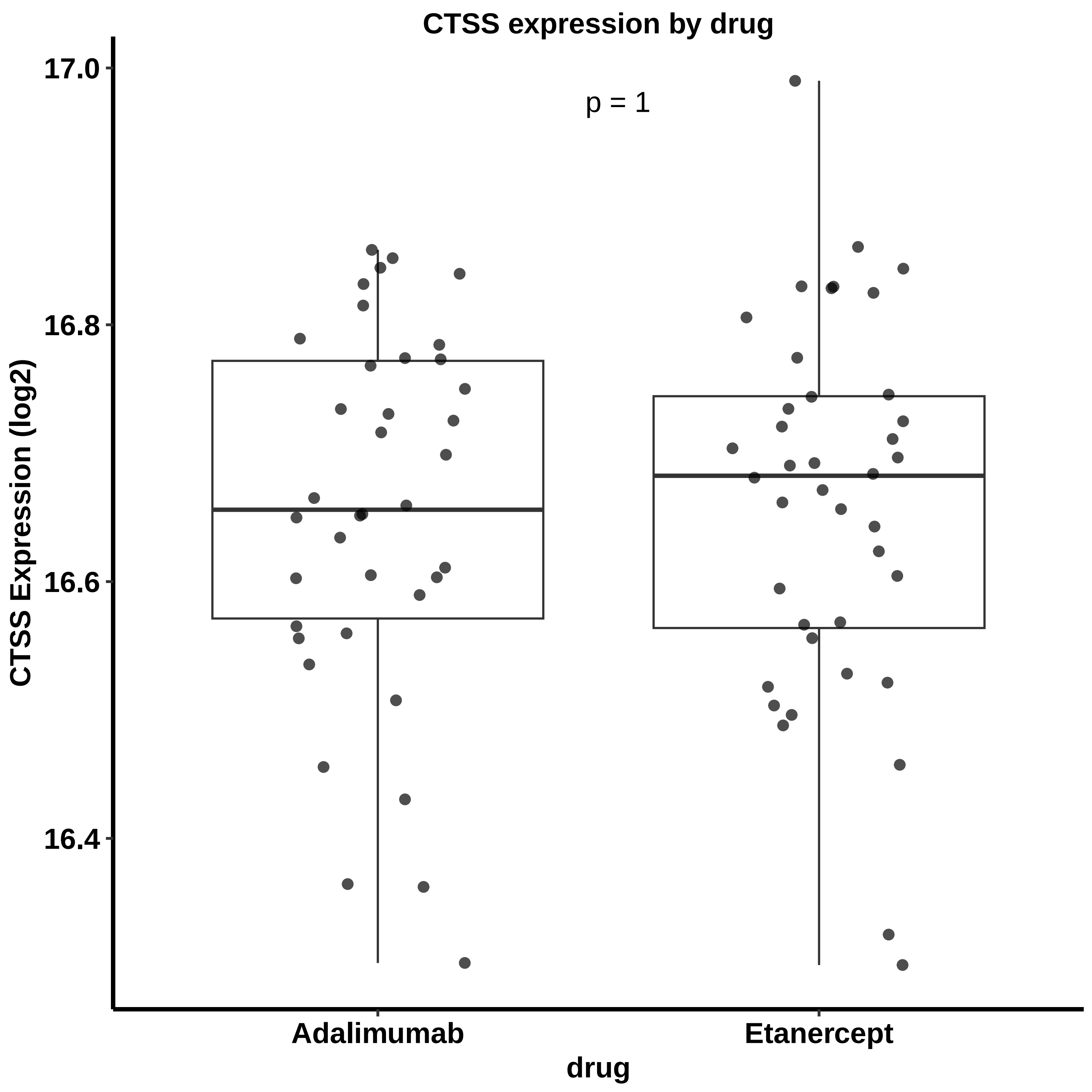

CTSS expression by drug
p = 1
17.0
16.8
16.6
16.4
CTSS Expression (log2)
Adalimumab
Etanercept
drug

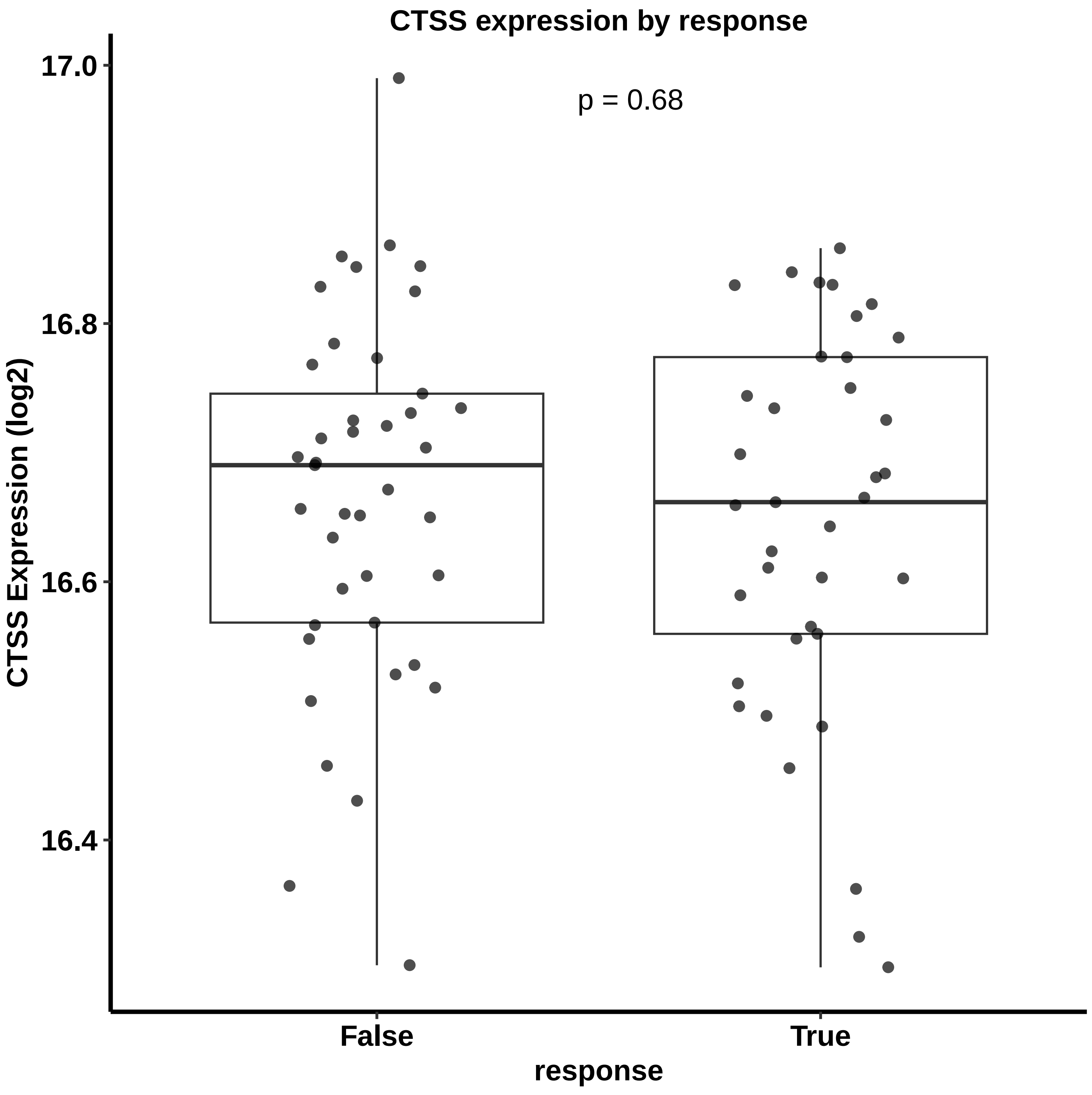
CTSS expression by response
p = 0.68
CTSS Expression (log2)
17.0
16.8
16.6
16.4
False
True
response

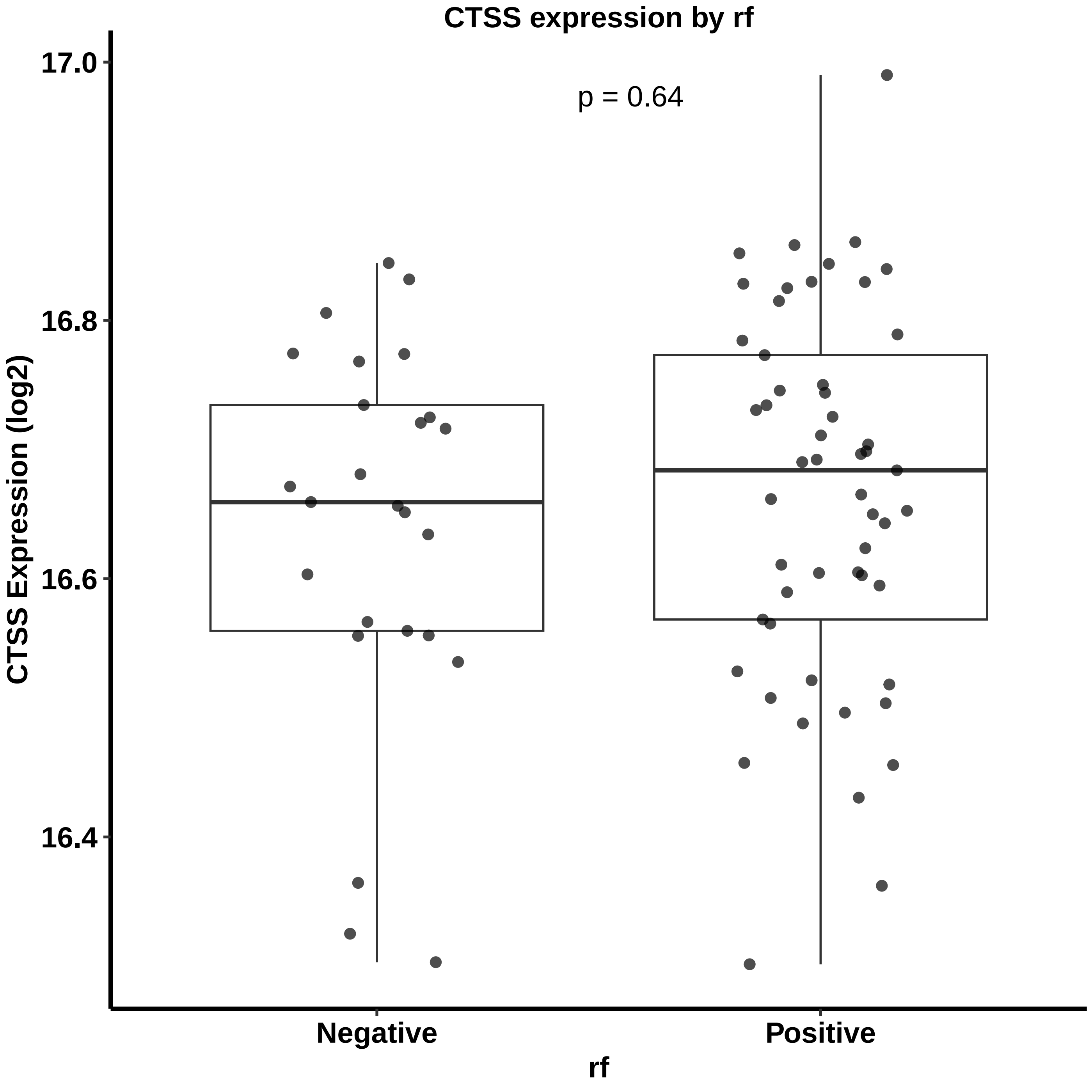
CTSS expression by rf
p = 0.64
17.0
16.8
16.6
16.4
CTSS Expression (log2)
Negative
Positive
rf

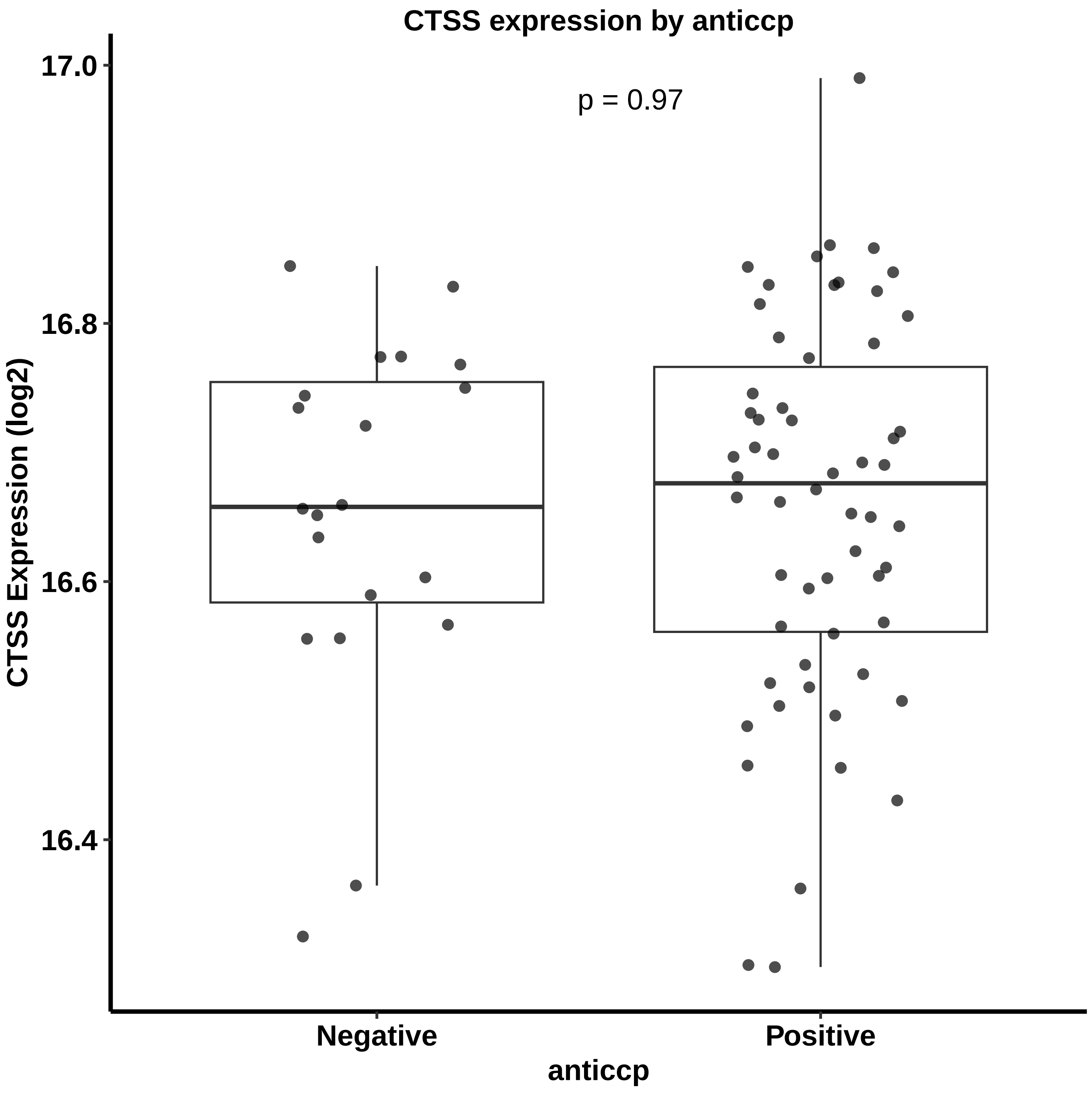

CTSS expression by anticcp
p = 0.97
17.0
16.8
16.6
16.4
CTSS Expression (log2)
Negative
Positive
anticcp

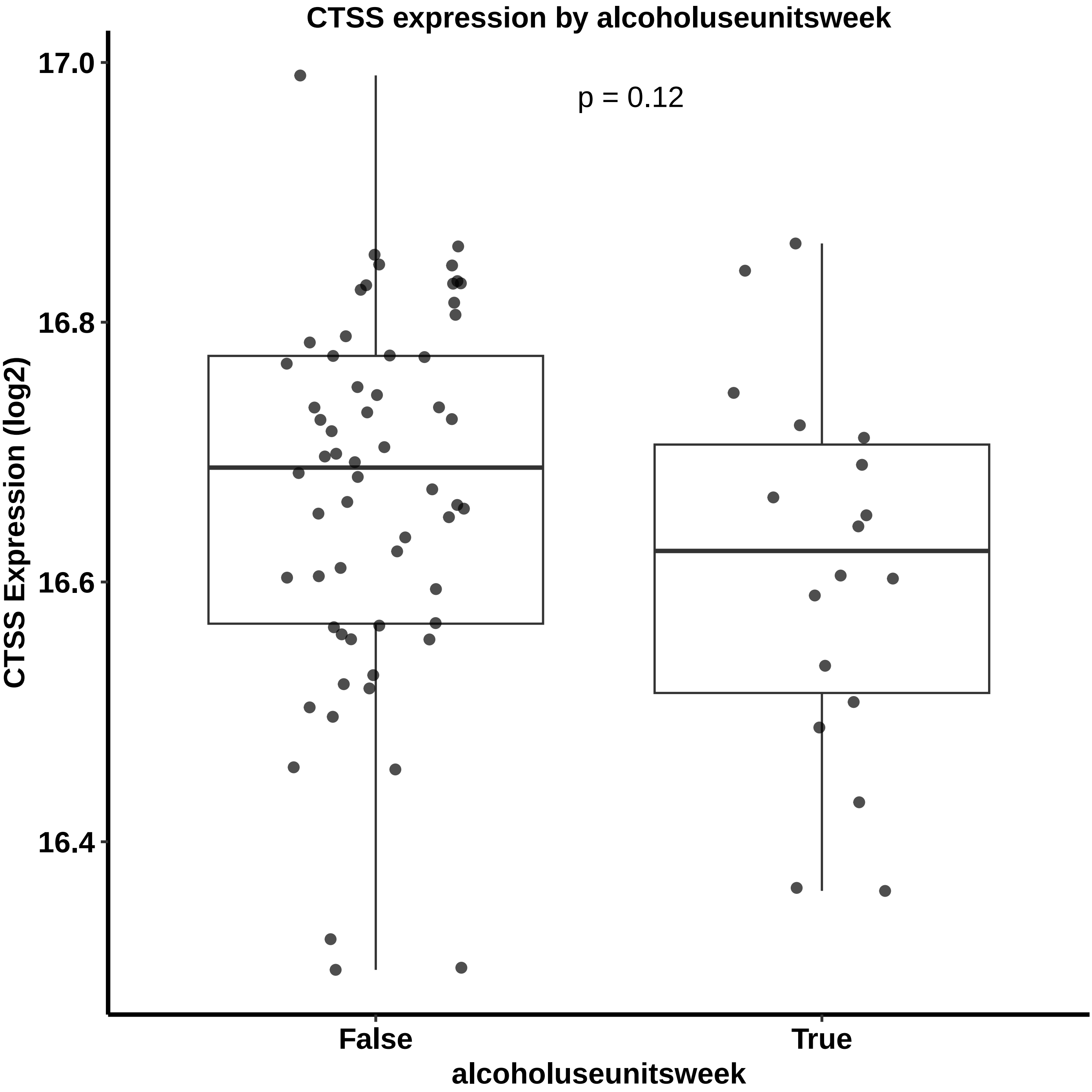

CTSS expression by alcoholuseunitsweek
p = 0.12
17.0
16.8
16.6
16.4
CTSS Expression (log2)
False
True
alcoholuseunitsweek

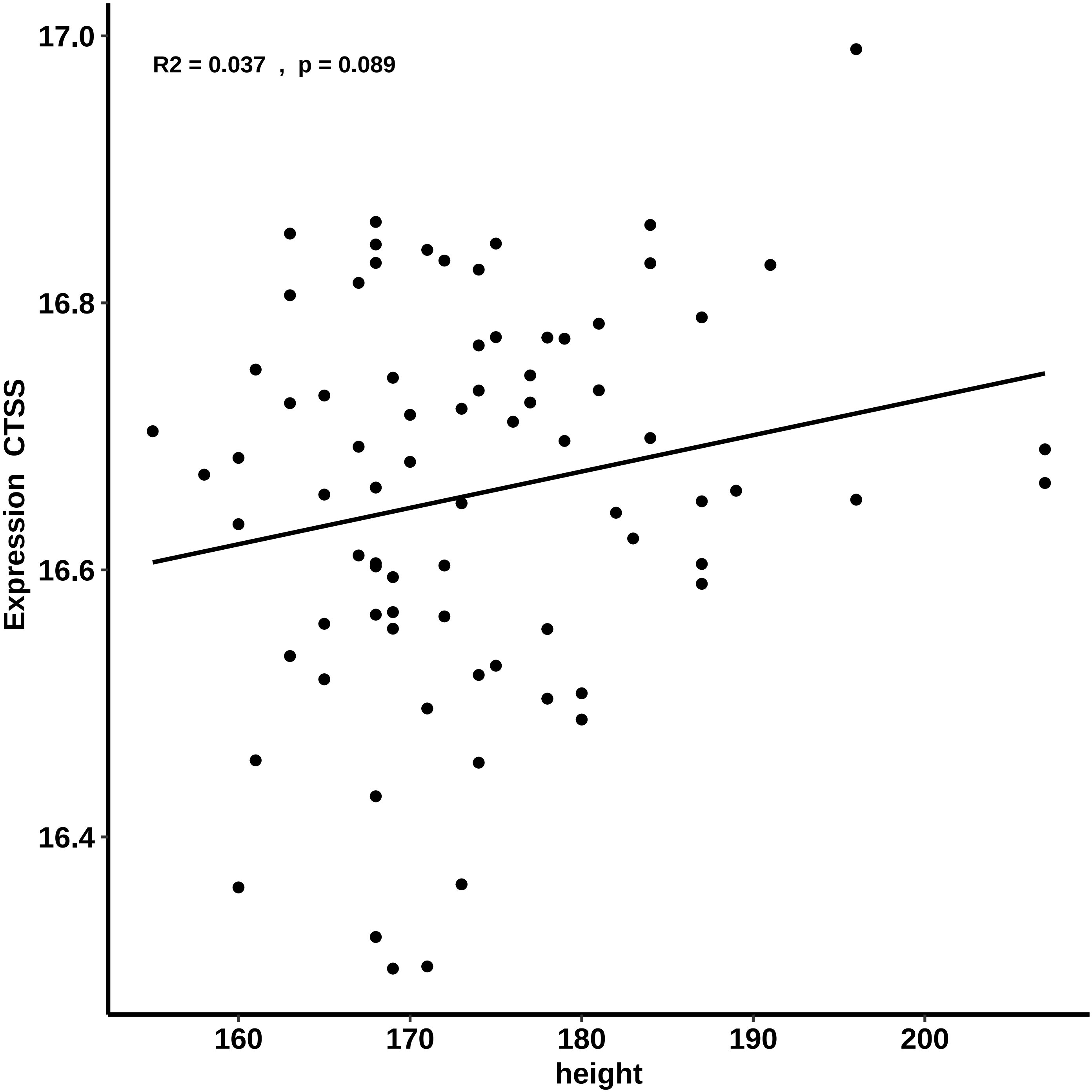

R2 = 0.037 , p = 0.089
Expression CTSS
17.0
16.8
16.6
16.4
160
170
180
190
200
height

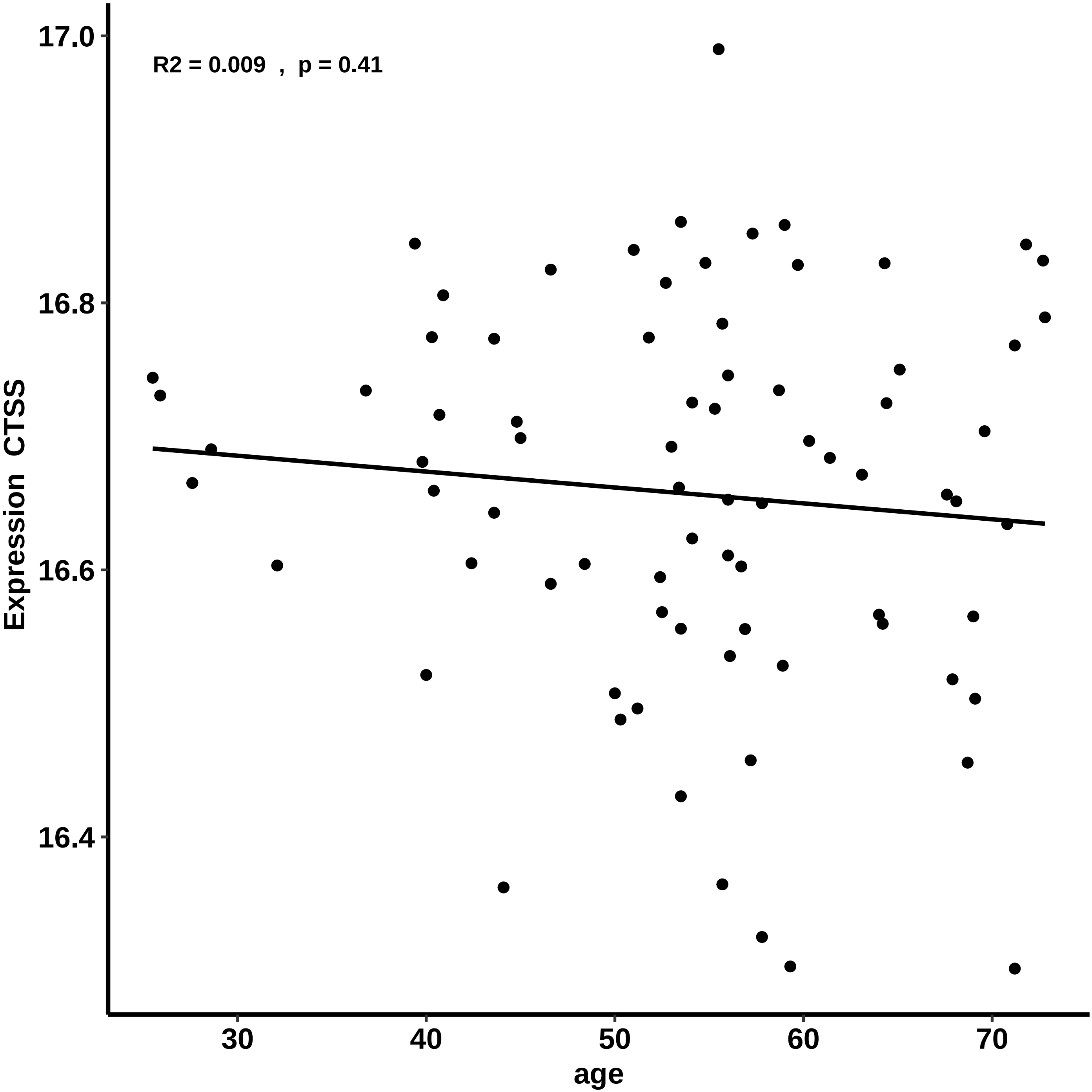
R2 = 0.009 , p = 0.41
17.0
16.8
16.6
16.4
Expression CTSS
30
40
50
60
70
age

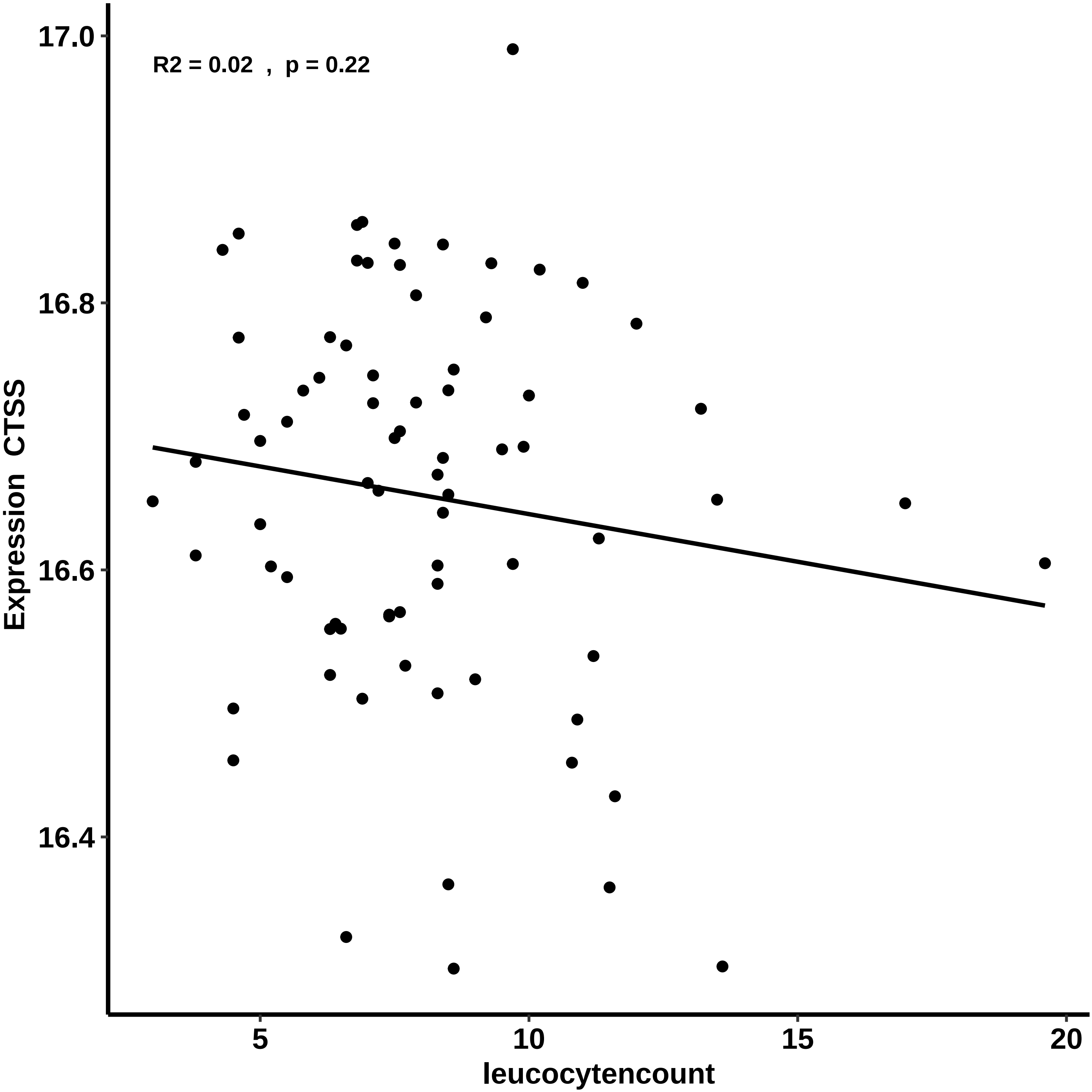

R2 = 0.02 , p = 0.22
17.0
16.8
16.6
16.4
Expression CTSS
5
10
15
20
leucocytencount

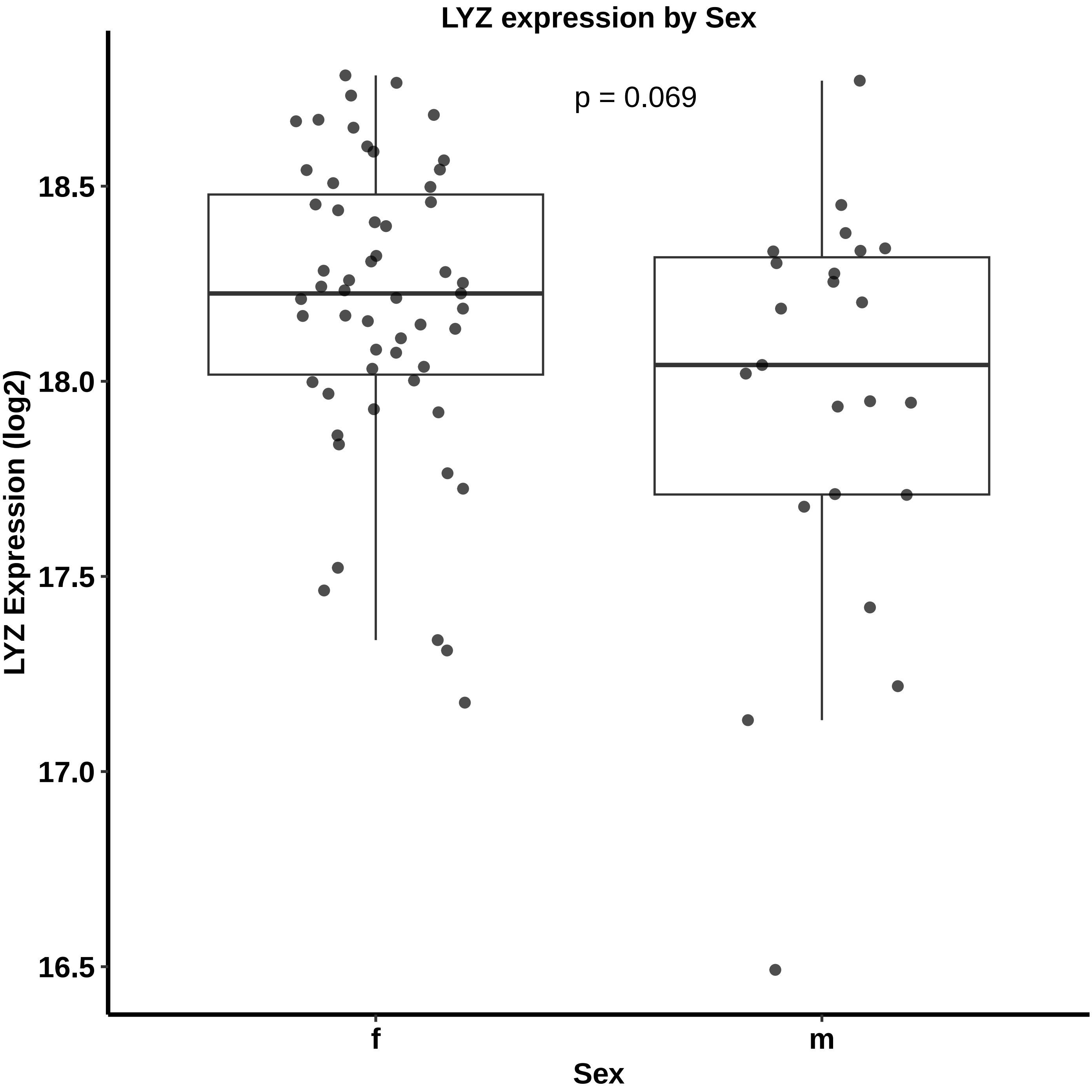
LYZ expression by Sex
p = 0.069
LYZ Expression (log2)
18.5
18.0
17.5
17.0
16.5
f
m
Sex

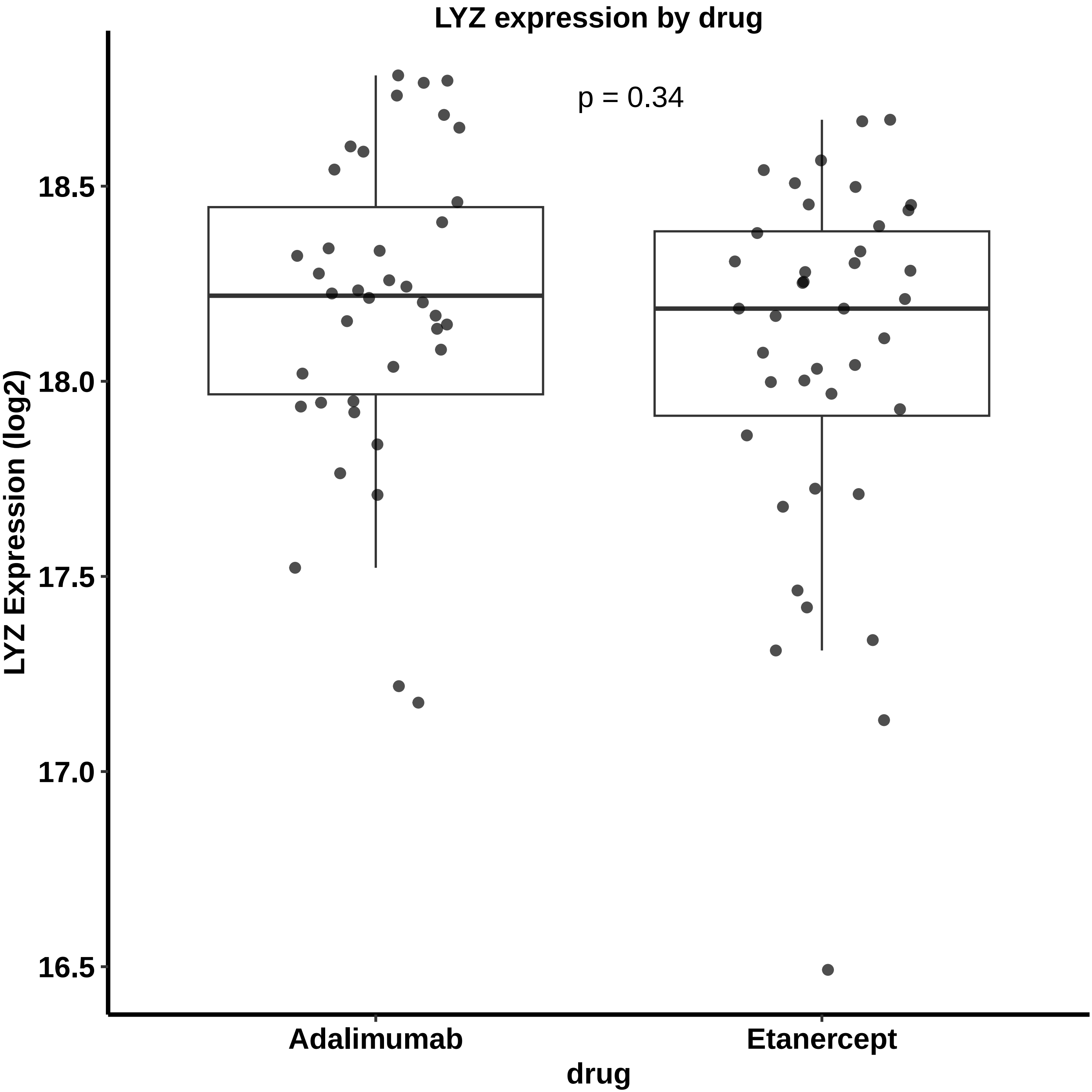
LYZ expression by drug
p = 0.34
LYZ Expression (log2)
18.5
18.0
17.5
17.0
16.5
Adalimumab
Etanercept
drug

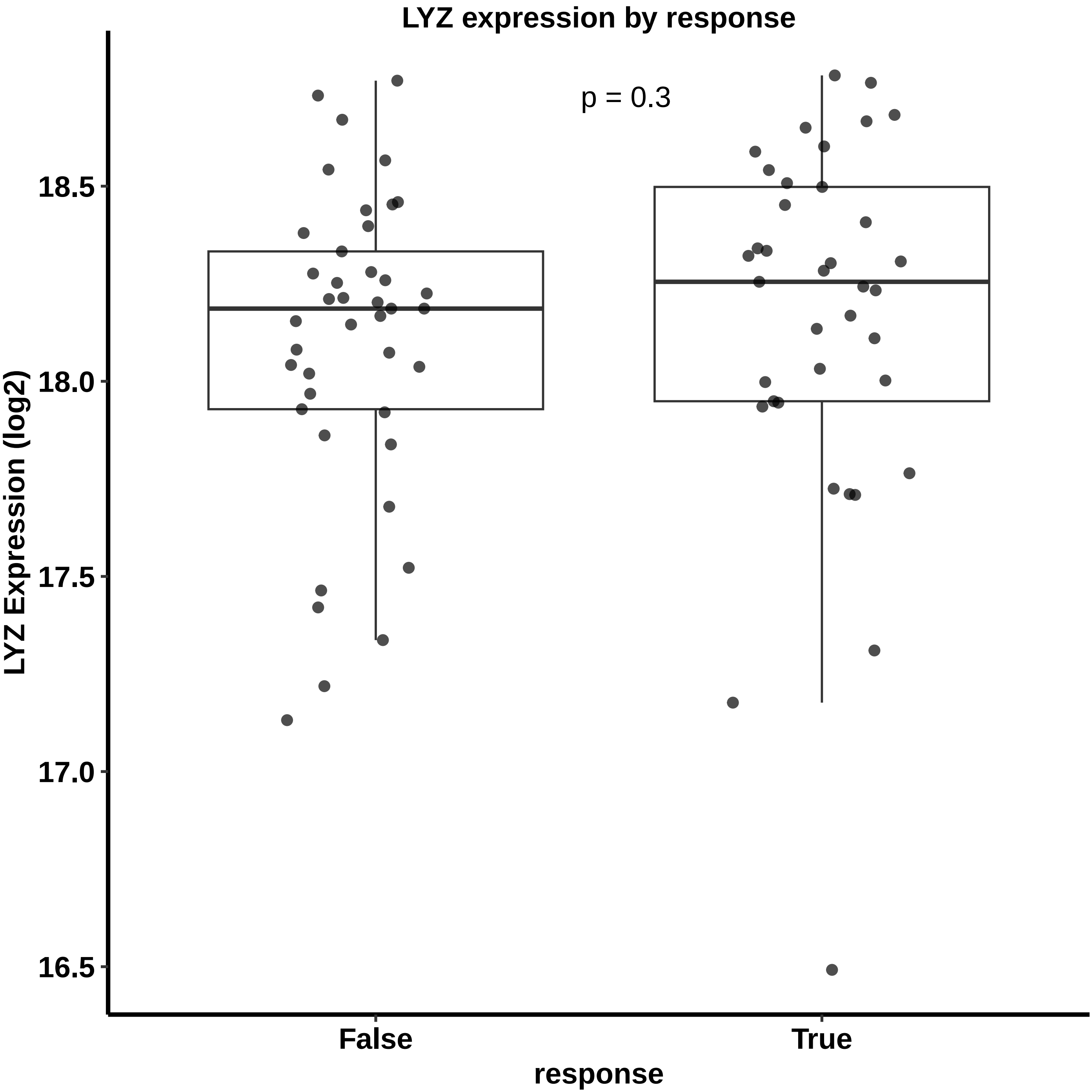

LYZ expression by response
p = 0.3
18.5
18.0
17.5
17.0
16.5
LYZ Expression (log2)
False
True
response

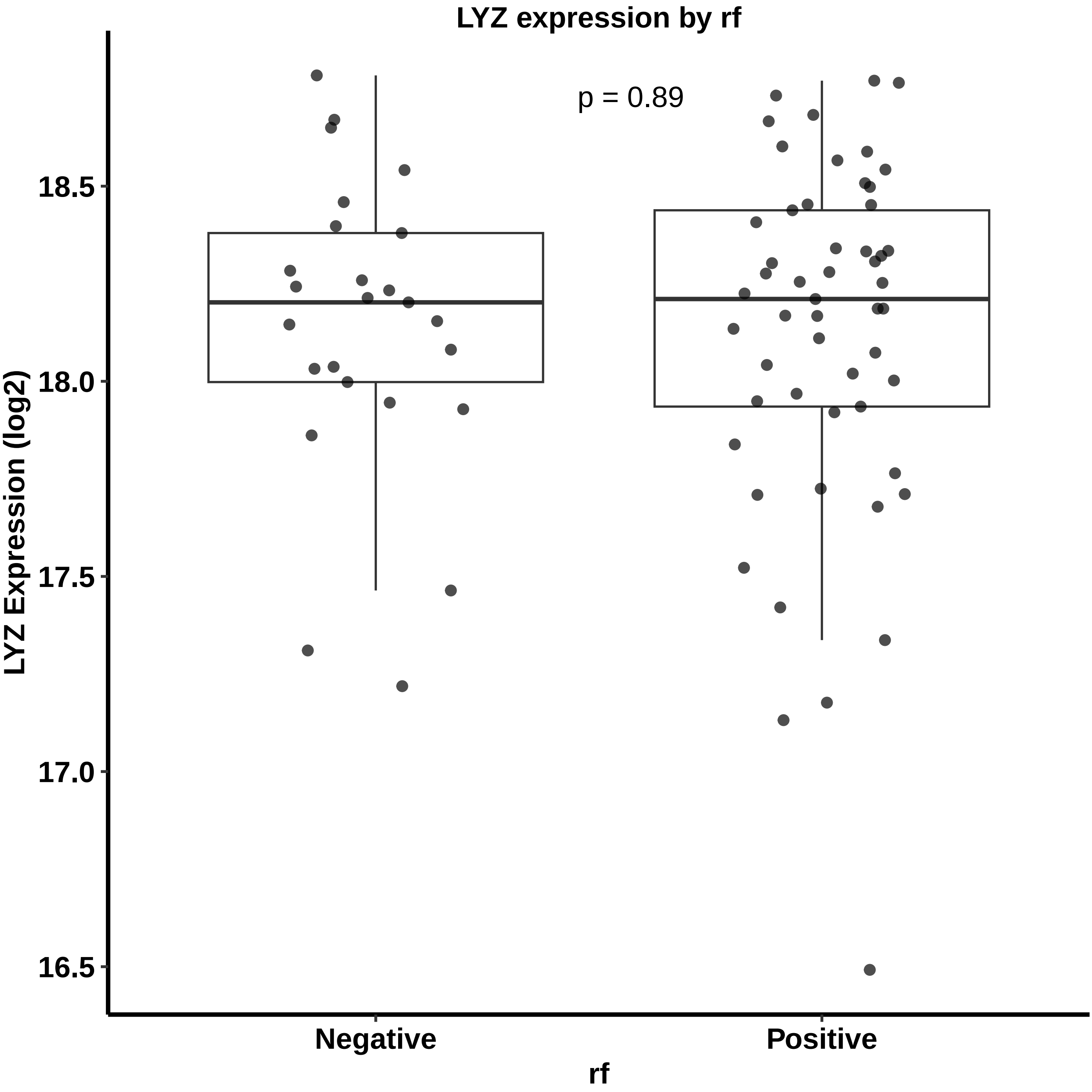
LYZ expression by rf
p = 0.89
18.5
18.0
17.5
17.0
16.5
LYZ Expression (log2)
Negative
Positive
rf

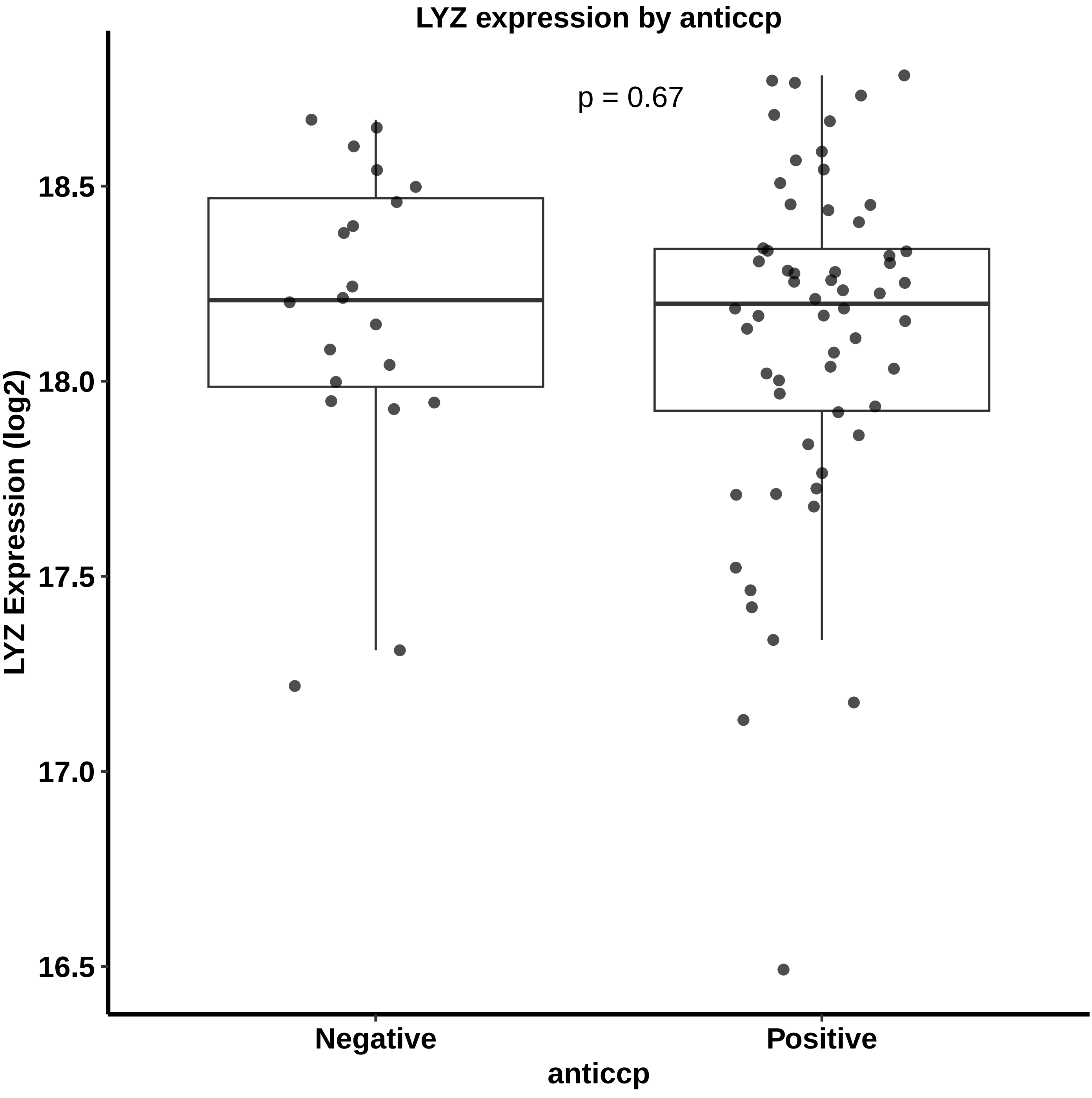

LYZ expression by anticcp
p = 0.67
LYZ Expression (log2)
18.5
18.0
17.5
17.0
16.5
Negative
Positive
anticcp

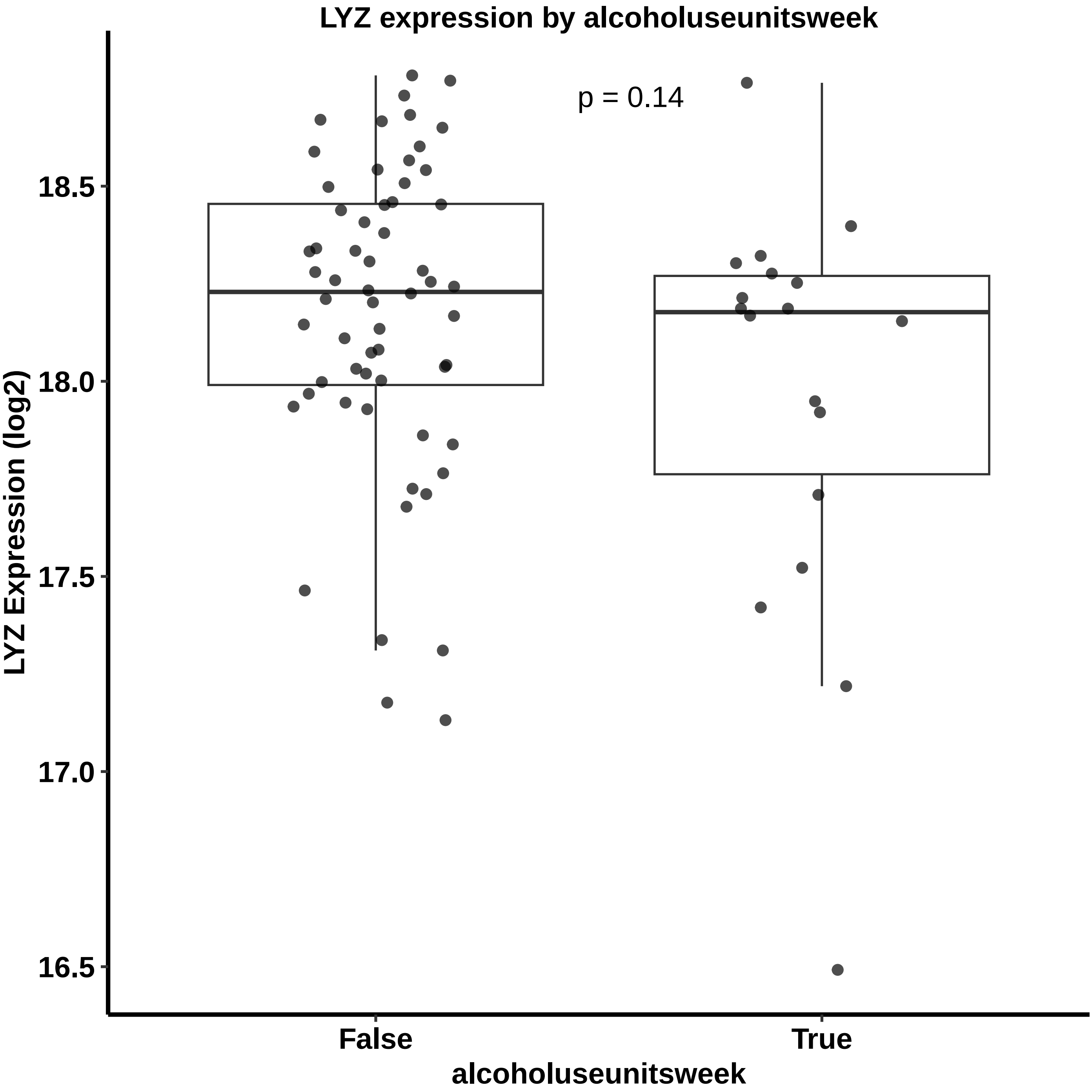
LYZ expression by alcoholuseunitsweek
p = 0.14
LYZ Expression (log2)
18.5
18.0
17.5
17.0
16.5
False
True
alcoholuseunitsweek

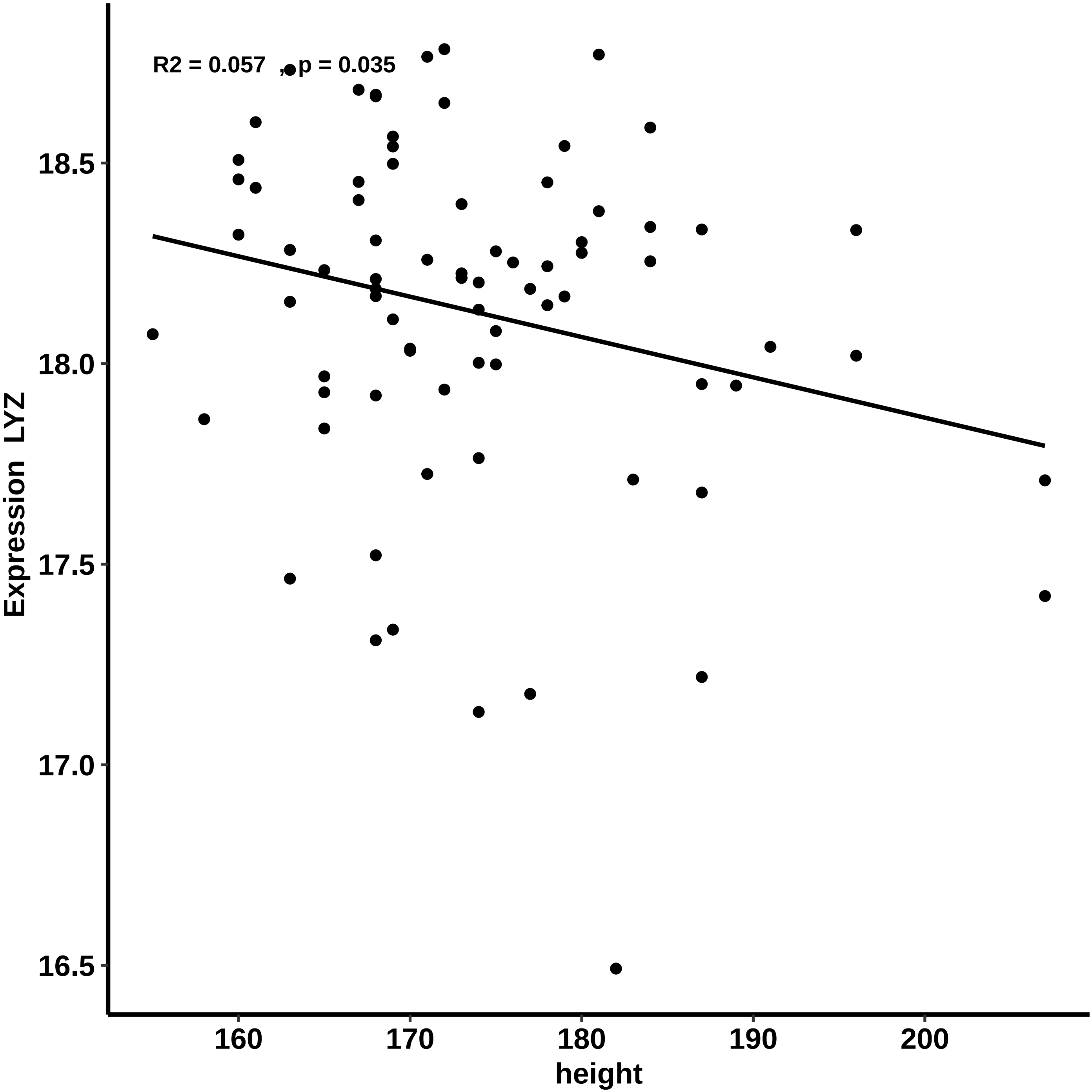

R2 = 0.057 , p = 0.035
Expression LYZ
18.5
18.0
17.5
17.0
16.5
160
170
180
190
200
height

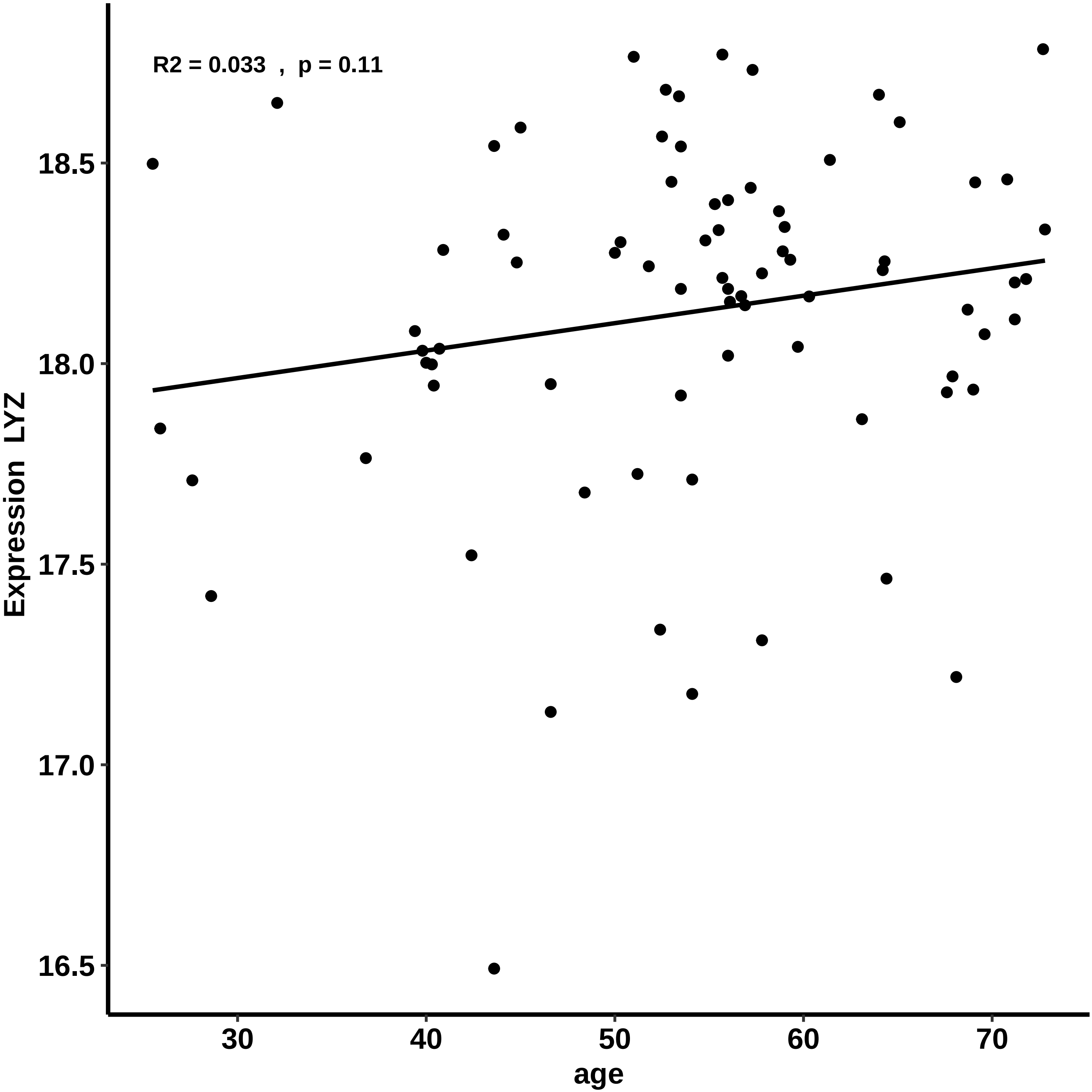

R2 = 0.033 , p = 0.11
Expression LYZ
18.5
18.0
17.5
17.0
16.5
30
40
50
60
70
age

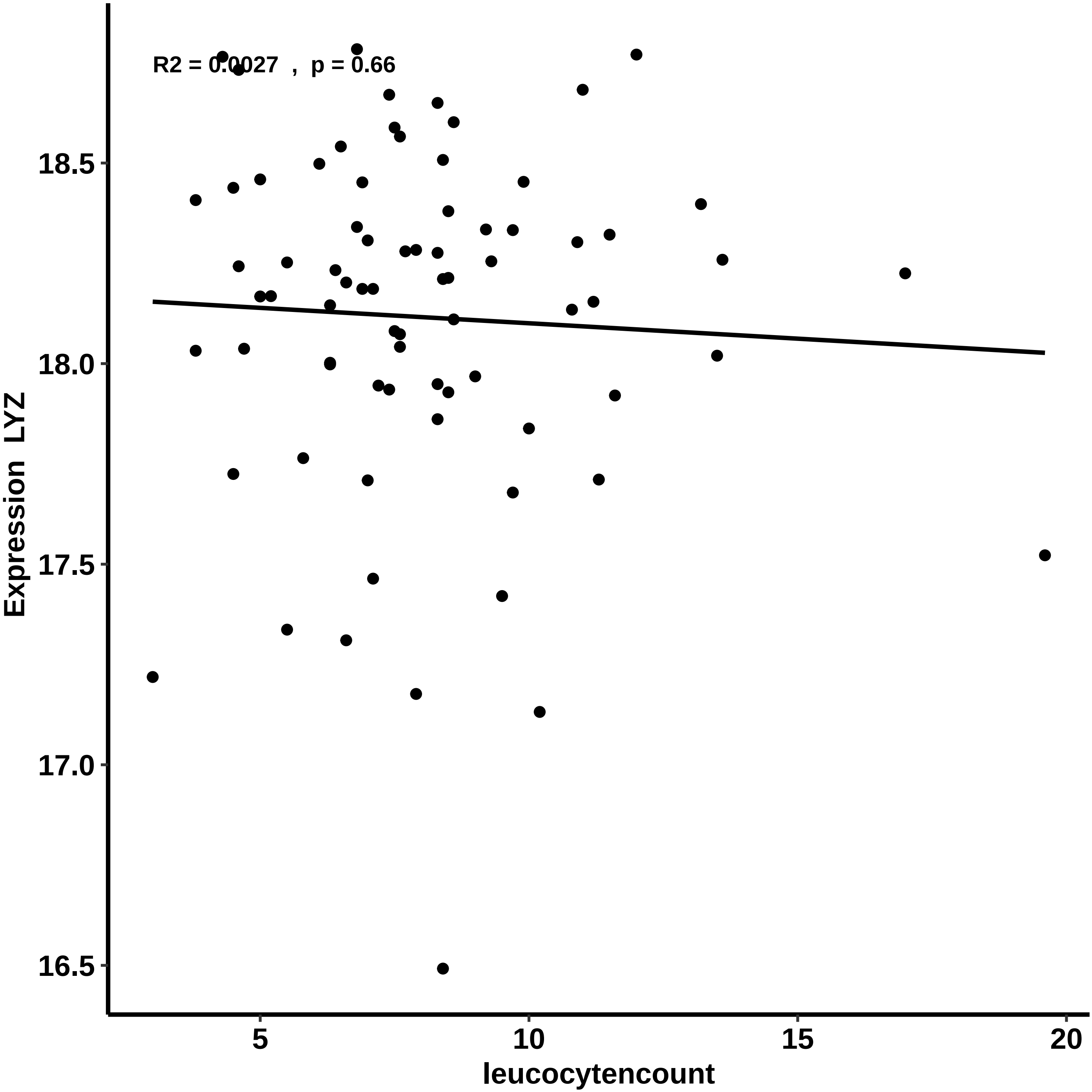
R2 = 0.0027 , p = 0.66
18.5
18.0
17.5
17.0
16.5
Expression LYZ
5
10
15
20
leucocytencount
